# The Liverpool Care Pathway for the Dying Patient: a critical analysis of its rise, demise and legacy in England

**DOI:** 10.12688/wellcomeopenres.13940.2

**Published:** 2018-04-24

**Authors:** Jane Seymour, David Clark

**Affiliations:** 1 School of Nursing and Midwifery, University of Sheffield, Barber House, Clarkehouse Road, Sheffield, S10 2LA, UK; 2 School of Interdisciplinary Studies, University of Glasgow, Rutherford/ McCowan Building, Dumfries, DG1 4ZL , UK

**Keywords:** Palliative care; end-of-life care; Liverpool Care Pathway; integrated care pathway; boundary object

## Abstract

**Background**: The Liverpool Care Pathway for the Dying Patient (‘LCP’) was an integrated care pathway (ICP) recommended by successive governments in England and Wales to improve end-of-life care. It was discontinued in 2014 following mounting criticism and a national review.  Understanding the problems encountered in the roll out of the LCP has crucial importance for future policy making in end of life care. We provide an in-depth account of LCP development and implementation with explanatory theoretical perspectives. We address three critical questions: 1) why and how did the LCP come to prominence as a vehicle of policy and practice? 2) what factors contributed to its demise? 3) what immediate implications and lessons resulted from its withdrawal?

**Methods**: We use primary and secondary sources in the public domain to assemble a critical and historical review. We also draw on the ‘boundary object’ concept and on wider analyses of the use of ICPs.

**Results**: The rapidity of transfer and translation of the LCP reflected uncritical enthusiasm for ICPs in the early 2000s. While the LCP had some weaknesses in its formulation and implementation, it became the bearer of responsibility for all aspects of NHS end-of-life care. It exposed fault lines in the NHS, provided a platform for debates about the ‘evidence’ required to underpin innovations in palliative care and became a conduit of discord about ‘good’ or ‘bad’ practice in care of the dying. It also fostered a previously unseen critique of assumptions within palliative care.

**Conclusions**: In contrast to most observers of the LCP story who refer to the dangers of scaling up clinical interventions without an evidence base, we call for greater assessment of the wider risks and more careful consideration of the unintended consequences that might result from the roll out of new end-of-life interventions.

## Introduction

Major policy innovations covering a whole jurisdiction are rare in palliative care. When one such intervention is introduced with government support and with a vigorous programme of implementation, but then runs into significant difficulties, it is vital to make sense of the factors at work. This paper draws on the concept of ‘boundary objects’ ^1^ to provide an in-depth analysis of an unsuccessful attempt to provide palliative care at scale. The Liverpool Care Pathway for the Dying Patient (LCP) was an intervention based on an integrated care pathway. It grew out of the hospice context and over more than a decade was promoted across the health care system in the United Kingdom before it was suddenly withdrawn from use. It has become a critical learning point for those charged with delivering end of life care to large populations and there is continued interest in trying to make sense of its rise and demise.

There is a wider context to such concerns. The importance of palliative care interventions to relieve suffering in the face of advanced disease and of death, has gained policy, clinical and academic endorsement worldwide ^2, 3^. Beginning in the 1960s with the emergence of new hospice programmes, the modern field of palliative and end of life care grew rapidly in the later decades of the twentieth century and has continued to make progress, attracting wide interest and support, and extending its reach ^4^. The World Health Organization first defined palliative care in 1990 ^5^ and in 2014 the World Health Assembly issued a resolution calling on all governments to adopt policies to support the delivery of palliative care across the life course and in all relevant institutional and community settings ^6^. Nevertheless, palliative care faces many challenges. It is still weakly developed in many low and middle income countries, and at the same time advanced and well-resourced health care systems are struggling to deliver its benefits, that have been demonstrated in specialist settings, across the wider spectrum of health and social care services.

The epidemiology of dying is also changing. Increasingly for many, death will follow an extended period of uncertainty, frailty and multiple morbidity in advanced old age. In contrast, the rapidly progressing downward trajectory of dying with its clear point of entry to the dying phase, which was central to the original hospice model of cancer palliative care, will become less common ^7^. Already a series of complex challenges is emerging around prognostication, communication and the planning of care for the gravely ill or dying person, especially in hospitals ^8^. The scenario has given rise to numerous attempts to take specialist palliative care knowledge and apply it ‘at scale’ within the mainstream of the health and social care system – in hospitals, care homes, and in the community. 

The LCP was a specific and concerted attempt to respond to these challenges. It was first described in a publication in 1997 ^9^ as a means of transferring key principles derived from hospice care into general health care settings, such as hospitals and care homes. It was endorsed by successive governments and in particular adopted as a key initiative within a National Programme and Strategy for End of Life Care in England and Wales ^10^. Then, in the face of mounting criticism and following a government requested national review, led by Baroness Julia Neuberger in 2013 ^11^, it was abruptly discontinued. An extensive debate ensued, seeking to understand how this had happened and why the LCP had ‘failed’.

Our purpose in subjecting the LCP story to detailed scrutiny is to move beyond the positions so far seen in the literature. These polarise between simplistic and retrospective ‘blaming’ of its limitations, often from sources that had hitherto been silent on the matter; or on the other hand, regret for its demise. Both these tendencies are evident in the many published commentaries, particularly in the clinical literature after 2013 ^12, 13^. In contrast, we seek to provide a theoretically informed analysis of the rise and fall of the LCP and to conclude with comments that might be relevant to future policy and practice in the context of complex health systems in which the character of terminal illness is undergoing significant change. We aim to answer the following questions: 1) why and how did the LCP come to prominence as a vehicle of policy and practice 2) what factors contributed to its demise? 3) what immediate implications and lessons resulted from its withdrawal?

## Background

### Integrated care pathways

Integrated care pathways ( Box 1) are complex interventions to enable the organization of health care for specific groups of patients, often in the context of time limited decision-making ^14^. Their use commonly involves structured documents outlining essential steps in care to be followed by members of multidisciplinary teams involved with particular groups of patients. They can also be used to introduce clinical guidelines and to provide a framework for audit ^15^. A key intent behind their use is to standardise or ‘rationalise’ care for a particular issue, problem or clinical care episode ^16, 17^, although their potential to enhance ‘person’ centred care is also often emphasised, creating a degree of ambivalence about their core purpose ^18, 19^.

**Box 1.** Integrated care pathways

Orientation • Multidisciplinary • Based on guidelines and evidence where available • For a specific patient/patient group • Forming all or part of the clinical record • Document the care given • Facilitate the evaluation of outcomes Components • A front page (paper or electronic) with patient identifiers, criteria, etc and a section for signatures • Protocol for use • Chronological plan for care • Details of guidance/ instructions • Variance recording section (allowing staff to record when a patient does not follow the usual or expected pattern for that care episode and the reason why). Based on information from the Scottish Pathways Association www.scottishpathways.com/what-is-an-integrated-care-pathway Accessed August 31st, 2017

Integrated pathways can go by various names: critical pathways, clinical pathways, and case management plans ^14^. Originating in the USA in the 1980s in the context of a ‘payment for service’ health care system, they were part of a wider movement to manage concerns about spiralling health care costs, whilst sustaining care quality and improvement. Their appearance in the United Kingdom (UK) in the 1990s, mirrored a ‘modernisation’ emphasis on clinical efficiency and a drive to ensure the application in everyday practice of standardised national guidelines ^20^. The integrative focus of pathways refers to the intent to use them as a means of formalising multidisciplinary channels of communication and of enabling professionals to work together across disciplinary or care setting boundaries (for example in health and social care) ^21^.

In a review of the evidence base behind integrated care pathways in stroke care, Allen and Rixson ^22^ note the widely cited aims of integrated pathways within NHS discourse: ‘…the right people, doing the right things, in the right order, at the right time, in the right place, with the right outcome’(p81). The reviewers conclude that integrated pathways in stroke care are potentially successful in the acute context, where the patient’s illness and care trajectory are reasonably predictable; however, their value in rehabilitative care, where recovery pathways are variable, is less clear. In a similar vein, Pinder *et al.* ^19^ describe how pathways ‘abstract the patient and reify the condition’ (p765), creating a tendency to ‘….omit the plasticity of patients’ personal circumstances and lived experience, providing no map of the terrain that the ill person has to traverse’ (p775). In this sense, pathways can have a powerful influence on shaping practice and the way in which practitioners understand clinical issues, and the most appropriate response to them.

An early review of integrated care pathways published in the *British Medical Journal* in 1998 ^23^ summarises them as ‘task oriented care plans’ offering not only the essential steps in patient care but also a structured means of implementing local protocols of care based on evidence based guidelines and analysing why care may fall short of, or vary from, any adopted standards.

In this context, the LCP was a clear example of an integrated care pathway which reflected all of these features, although as we will see, debate later emerged about the quality of evidence on which its key features were based and whether it was effective in achieving its goals. Such issues were foreshadowed in an observation made from the 1998 review: ‘…despite the sound principles which underlie care pathways, few evaluations have been done of the cost of developing and implementing them and their effectiveness in changing practice and improving outcomes’ ^23^ (p133).

### ‘Boundary objects’

A boundary object is an artefact that provides a means of sharing ideas, technologies and practices across and between organisational settings, cultures and communities ^24^. The interest in boundary objects reflects a concern to articulate the meanings and perspectives of actors from a variety of ‘social worlds’ or ‘sites of difference’ ^25^. Originally conceptualised in 1989 by Star ^26^ as ‘…objects which both inhabit several intersecting social worlds … and satisfy the informational requirements of each of them’ (p393), boundary objects were soon categorised along four dimensions: repositories, ideal types, maps and standardised forms ^1^. Integrated care pathways have been described by Allen ^27^ as ‘classic examples’ (p305) of boundary objects because they straddle clinical, managerial and user interests. They are thus potentially associated with differing meanings across the particular groups involved.

Following Carlile’s ^28^ definition of a successful boundary object as one which provides a shared language, allows concerns to be expressed and enhances knowledge, Fox ^29^ has made the distinction between ‘positive’ and ‘negative’ boundary objects, each of which have consequences for the transformation of knowledge and practice within a community. This highlights examples where implementation is able to proceed or to where it is blocked in some way. Both are important.

Boundary objects are therefore subjects of and for reinterpretation and renegotiation, often through a process of conflict, although their dynamic aspects have been somewhat neglected ^30^. While boundary objects are capable of bridging different perspectives, they can be associated with negative unintended consequences resulting in inhibition of the very improvements they were intended to facilitate ^30^. For Carlile, the production of knowledge across boundaries entails several activities: transfer (involving information and knowledge processing), translation (involving interpretation and the use of new information and knowledge), and transformation, which occurs when the interests of actors diverge, leading to power struggles over the legitimacy of the ‘object’ at hand ^28^.

### Methodological orientation

Building on the twin concepts of integrated care pathways and boundary objects, we set out to write a critical and historical account of the LCP, informed by relevant theory and thereby generating understanding that may inform future end of life interventions. We have made use solely of sources available in the public domain, namely:

1) Content emerging from the LCP programme – this includes guidance materials, LCP documentation, and writings concerned to support the use of the LCP.

2) Evidence from a spectrum of published studies on the use of the LCP – qualitative research and improvement project findings, surveys of practice and attitudes, clinical evaluations, and randomised trials.

3) Letters, articles, broadcasts, and online content found in the mass media and on social media.

4) Professional commentaries on the LCP, particularly those written in the aftermath of its withdrawal and published in clinical journals.

5) Content from and associated with the Neuberger report ^11^ into the use and efficacy of the LCP.

We contend that these sources are sufficient to answer our three research questions. We acknowledge that other lines of enquiry about the LCP are still to be pursued, and that these would require other methods. For example, oral history interviews might be useful in assessing the rise and demise of the LCP from the personal perspectives of key actors involved in the wider processes we describe in this paper. We suggest that it may still be too early for such work to be conducted, as personal investments in the LCP, individual and organisational reputations, questions of anonymity, confidentiality, and other sensitivities may be sufficiently marked as to inhibit the successful conduct of such work. Primary and secondary sources in the public domain still offer much material for analysis however, and allow the short to medium term barriers that would arise from research involving human subjects, to be overcome.

## History and development

The LCP was originally formulated during the 1990s at the Royal Liverpool University Hospitals NHS Trust and the Marie Curie Hospice in Liverpool and underwent incremental development and revision over the next 15 years ^31^. The LCP was subject to annual review by a multi-disciplinary steering group that included carer representation. Utilising the quality improvement methodology of ‘Plan, do, study, act’ (PDSA) ^32^, the LCP programme and documentation was updated and revised over time in response to feedback, with 12 versions published in total.

The LCP was focused on patients in the ‘dying phase’ (described as the last 48 hours of life in the original LCP documentation). Up to version 11, recognition of the dying phase was recommended to be the responsibility of the multi-professional team agreeing that the patient was dying and when at least two of the following features were present: the patient is bedridden; the patient is semi-comatose; the patient is able to take only sips of fluids; the patient is no longer able to take tablets ^9^. Following review of feedback by the multi-disciplinary steering group, version 12 removed these features and replaced them with an algorithm to guide recognition of the dying phase.

Once established that the patient was entering the dying phase, the use of the LCP entailed the following four steps: initial assessment; care planning against suggested ‘goals’ of care; ongoing assessment and care after death ^33^. The documentation associated with the LCP was intended to replace all other medical and nursing notes in use and was designed to prompt and guide clinical decisions and interventions for the dying patient, in anticipation that it would aid good communication with family members and the patient, and improve the quality of end of life care ^34^. It took the form of a ‘template’ for use by the various clinicians involved in a person’s care, addressing four domains of care: physical, psychological, social and spiritual ^35^. It gave space to record clinical decisions and actions, together with prompts and guidance on the different aspects of care required. These included: comfort measures, anticipatory prescribing of medications, discontinuation of inappropriate interventions, and the psychological and spiritual/existential support of the patient and family ^35^. An example of LCP documentation indicating initial assessment of the patient and addressing the first goal of care is provided in Supplementary File 1, drawing on the last generic version 12, published in 2009.

Perhaps in anticipation of the risk that the LCP could be seen as a set of instructions for care rather than a guide to individualised decision-making, the importance of ‘variance recording’ was emphasised (see Box 1). For example, in their text book about the LCP, Ellershaw and Wilkinson noted that variance recording in the context of integrated care pathways is:

…a mechanism by which a seemingly process driven approach to care can be tempered in line with individual patient need. The potential to use clinical skill and judgement to deviate from the suggested plan of care in response to an individual patient’s needs makes the LCP a more flexible and practical document. Variance recording tells the story of the patient’s journey and current condition ^35^ (p17).

The context of the development of the LCP was twofold: first, recognition that NHS hospital care of the dying fell short of best practice as understood at the time, and second, a recognition that the shortfall in specialist palliative care resource in hospitals meant it was unrealistic to expect specialist palliative care teams to be involved with every patient ^9^. The LCP was therefore about ‘going to scale’ with an approach that had to date been limited in its availability.

## Transfer

The Marie Curie Palliative Care Institute in Liverpool took the lead role in disseminating information about the LCP, through series of publications ^34, 36– 38^ and a process of networking nationally and later internationally. The work was co-ordinated by a central LCP team and organised in a series of programmes or work streams. In a 2005 publication Ellershaw and Murphy describe the extent of this enterprise and how it worked:

The LCP framework is now based at the Marie Curie Palliative Care Institute Liverpool and is focused on four programmes; these are non-cancer, bench marking, education and international. These four programmes are supported by an audit and a research team. The education programme for 2005 will incorporate 2500 health-care professionals. Five clinical champions and 13 clinical facilitators now support the central team in the education-spread programme. An annual national conference based on the theme of care of the dying acts as a focus for new developments and research in this field ^39^ (p133).

The LCP had become a major enterprise with its own dissemination and transfer needs. There was a great deal to be done to inculcate LCP knowledge and capacity at a local level. The central team encouraged a six to 12 month local implementation process for the LCP, described by Murphy in 2003 as entailing ten steps (see Box 2). This later became known as the ‘Ten step continuous quality improvement programme’ or CQUIP and was described as occurring in four phases: induction, implementation, dissemination and sustainability ^40^.

**Box 2.** Recommended local implementation process for the LCP

1. Establishing the project, i.e. gaining executive and multidisciplinary endorsement for the LCP project 2. Development of documentation 3. Retrospective audit of current documentation 4. Induction — education programme 5. Implementation — education programme 6. Reflective practice 7. Evaluation and training needs analysis 8. Maintenance of education programme 9. Training the teachers 10. Programme of ongoing feedback from analysis of LCP Source: 40

### Wider endorsement

In 2000, a National Cancer Plan ^41^ was published by the government as part of its NHS modernisation programme, in which it was stated that one aim was to ‘improve the care of the dying to the level of the best’ (para 7.21). Implicit here was that such care, as delivered across hospitals and nursing homes, would be elevated to standards more typically found in the specialist settings of hospices and palliative care units. The following year, 2001, the LCP was recognised as good practice by an NHS ‘Beacon Programme’ launched in 1999 to identify services making significant contributions to the modernisation initiative ^42^. This was followed in due course by an announcement on the 26 ^th^ December 2003 that an NHS End-of-life Care Programme (later known as a National Programme and referred to here as ‘the Programme’) was to be established in early 2004 with funds of £12 million over three years to support the implementation of best practice in end of life care by widening the pool of trained staff ^43^. The LCP was prominently identified by the Programme ^44^ as one example of how this could be done, citing influential guidance published by the National Institute for Clinical Excellence (NICE) in 2004 ^45^ (para 8.33).

In addition to the LCP, two other ‘tools’ were recommended by the Programme: the Gold Standards Framework (to improve end of life care in primary care) ^46^, and Preferred Priorities for Care (an advance care planning intervention) ^47^. The three ‘tools’ together quickly became the main focus of an implementation project, devolved to 28 Strategic Health Authorities created by the government in 2002 in order to manage the NHS locally in England. For the Programme, the authorities were charged with identifying clinical priority groups and targeting care settings to work at local level. A small National Support Team, comprising a Programme Director and Programme Administrator was also established to support the SHAs for the duration of the Programme ^48^. In an early report from the Progamme, the LCP is described thus:

The Liverpool Care Pathway for the Dying Patient (LCP) was developed to take the best of hospice care into hospitals and other settings. It is used to care for patients in the last days or hours of life once it is known that they are dying. The LCP involves prompting good communication with the patient and family, anticipatory planning including psychosocial and spiritual needs, symptom control (pain, agitation and respiratory tract secretions) and care after death. The LCP has accompanying symptom control guidelines and information leaflets for relatives ^44^ (p2).

Use of the LCP received further high level endorsement in 2005 from the National Council for Palliative Care, with publication of a ‘Palliative Care Manifesto’ ^49^ in which one of four key pledges was to to introduce monitoring of care of the dying as a key element of performance management for NHS organizations at board level. Ellershaw and Murphy ^39^ note that, in addition, a report on cancer care from the National Audit Office ^50^ highlighted the role of the LCP in the National End of Life Care Programme as a:

…means of integrating care for the dying by pulling together different professional groups and providing a framework to help busy staff ensure the completeness of care procedures ^39^ (p133).

Most notably, it was recommended by the landmark National End of Life Care Strategy, published in 2008:

….[Trusts are] strongly recommended to ensure that the LCP is adopted and its use audited in all locations where patients are likely to die ^10^ (p 67).

By this point, the LCP as boundary object seems to have become firmly established: strategically, clinically, organisationally.

## Translation

The LCP was not a fixed entity. It went through a series of revisions as its nationwide use began to quicken. Its first iteration was reported in 1997, in the European Journal of Palliative Care ^9^ and the final ‘generic’ version was issued (version 12) in December 2009. The latter followed two years of consultation across the sector by the Marie Curie Palliative Care Institute, examination of two rounds of national audit data ^a^ ^51, 52^ and consideration of criticisms that were by now emerging in the media about risks associated with the use of the LCP ^53^. An associated ‘data dictionary’, perhaps seeking to reassure, provided detailed instructions about the use of the LCP to enable:

…explicit and robust understanding of the core meaning of each of the goals of care and the rationale, required behaviour and correct coding of information ^31^ (p4).

National and locally produced documents associated with version 12 also affirmed that:

…the responsibility for the use of the LCP generic document as part of a continuous quality improvement programme sits within the governance of an organisation and must be underpinned by a robust education and training programme ^54^ (p4).

By 2011, the LCP had received endorsement in a series of policy documents, including a report on quality markers and measures in end of life care ^55^ and in end of life care guidance issued by National Institute for Clinical Excellence ^45^ and the General Medical Council ^56^.

### Peer reviewed evidence and a developing critique

As the LCP became ‘high profile’ in the policy arena of end of life care in the UK, and especially so in England, it began to receive more attention from the academic research community and to be studied in other countries (notably the Netherlands). A key research criticism was that the LCP lacked an under-pinning ‘gold standard’ of evidence in the form of supporting data from randomised controlled trials (RCTs). The critique missed an important nuance: the LCP was styled quite explicitly as a quality improvement programme; something not usually associated with the more formalised approach of the RCT. This type of criticism was voiced openly in 2005, with publication of a letter in the journal *Age and Ageing* by a hospice doctor from Kent, Shah ^57^, lamenting the absence of large scale RCT data to support the LCP. Shah was writing in response to an earlier research letter published in 2004 by Ellershaw’s team ^58^ that reported descriptive data on use of the LCP on an acute stroke unit from a ‘before and after’ study of 20 clinical cases, which showed:

…marked improvement in levels of documentation, including a change in the prescribing of medication ^58^ (p625).

In response, Shah raised the following criticisms:

The LCP appears to have a potential to improve some of the patient and carer-centred outcomes of good death, but it would be helpful to have some evidence. ... Current circumstances almost resemble a setting of a multi-centre randomised trial in regards to LCP implementation. In hospitals of different sizes and variable set-ups there are wards implementing LCP, and others that are not. It will be useful to compare the patient-, carer- and staff-related outcomes of care of dying in intervention (LCP) and control (conventional care of the dying in hospitals) groups before the LCP is rolled out to more wards and hospitals. If we miss this opportunity, we will end up with an untested LCP accepted as a gold standard everywhere. We will then be unable to test its efficacy, as ethical approval will almost be impossible ^57^ (p197-8).

There is a reply to Shah by the LCP team in the same issue of *Age and Ageing* ^59^, taking the line that the whole field of palliative care is evidence poor and also observing that the LCP is a template of care that:

… promotes the spread of the palliative care approach in the dying phase to members of the generic team. In this way, it has the potential to impact on the ‘culture’ of the delivery of care to dying patients in a way that a relatively small team of palliative care professionals … could probably never do (p198).

Over the next four years, Ellershaw and his team published a number of observational studies on the LCP broadly located within the quality improvement paradigm ^60– 63^ ^b^. Over a similar time period, five Dutch papers were published reporting the results of non-controlled studies in the Netherlands ^64– 68^. Key findings are summarised below. In 2010 a Cochrane review was published: ‘End-of-life care pathways for improving outcomes in caring for the dying’ ^69^ looking for evidence about the LCP from randomised controlled trials: it found no studies to include.

In 2008, a flurry of correspondence was published in the *British Medical Journal* (BMJ) about the LCP, which foreshadowed some of the criticisms that later emerged in the public sphere. The correspondence was prompted by a controversial article describing the practice of continuous deep sedation until death in the Netherlands ^70^. It was initiated by Adrian Treloar, an old age psychiatrist in London, who accused the LCP of being ‘…the UK’s main clinical pathway of continuous deep sedation’ and voiced considerable doubts about its suitability for use among frail older patients because of the uncertainty of their typical dying trajectories. His letter prompted a robust response from Ellershaw, in an editorial seeking to correct ‘… dangerous misconceptions about the purpose and use of the LCP’ ^71^. Other letters in support of the LCP were also published ^72– 74^. Treloar subsequently apologised for his association of the LCP with continuous deep sedation ^75^, but persisted with his critique of the suitability of its criteria of use for frail older patients. Various aspects of Treloar’s critique were supported by other correspondents from geriatrics ^76^, general practice ^77^ and hospice care ^78– 80^. One contribution ^81^ was from a retired British geriatrician, Gillian Craig, who was at that time the Vice Chair of the ‘pro-life’ Medical Ethics Alliance ^c^. Craig supported Treloar’s original description of the LCP in relation to continuous deep sedation, and mirrored the content of her own opinion piece warning of the dangers of rolling out palliative care at scale, which had been published in the official journal of the American Academy of Hospice and Palliative Medicine ^82^.

In 2011, an integrative review ^83^, examined peer reviewed research about the LCP, published from November 2009 to April 2010. Articles were selected if an end-of-life care pathway was used to manage the dying phase in the acute care and/or hospice setting and if care delivered to dying patients and/or their families was evaluated. Articles were excluded if they reported a single case study or described process measures only. The review addressed five questions:

1) In which population(s) has the end-of-life care pathway predominately been used to manage care of the dying?
2) Is there evidence to support the end-of-life care pathway’s use in acute care and/or hospice systems?
3) What are the implications of these findings for evidence-based care of the dying in the acute care and/or hospice setting?
4) What are the key elements underpinning effective implementation of the end-of-life care pathway?
5) What are the gaps in the evidence and future research directions?

No randomized controlled trials or meta-analyses were identified, although 26 studies of other types ^d^ were included: 15 from the UK; four from the Netherlands; three from the USA; two from Australia and one each in Ireland and China. Phillips *et al.* ^83^ concluded that there was some low level evidence suggesting that: pathways promote good practice in end of life care; increase accessibility in palliative care and promote better management of patient comfort. However, they observed a range of weaknesses of end of life care pathways from their review, including: a lack of association with palliative care approaches that are ideally in place before the dying phase and dependence of use upon timely recognition and diagnosis of dying in a context where only around 50% of deaths in acute care settings are predicted ^83^.

Phillips *et al.* ^83^ also provided evidence from several studies of pathway implementation, indicating that key factors for successful implementation include: clinical education sessions and professionals with the necessary competences to use the pathway; strong clinical leadership; and the use of pathway ‘facilitators’. The criticality of facilitators to the safe use of the pathway for patients and the development of palliative care capacity amongst health care professionals was especially emphasised, though the review noted that there had been a decrease in the numbers of pathway facilitators in the UK over time:

The palliative care capabilities of the pathway facilitator appear to be central to ensuring that the dying patient’s transition onto the pathway is appropriately negotiated and safely managed. The pathway facilitator also plays a key role in building the palliative care capacity of health professionals. Despite this positive relationship, the number of U.K. pathway facilitators actually decreased over time, reflecting a trend to use facilitators for a defined period during the path-way implementation phase ^83^ (p952).

In response to the growing disquiet over the LCP, Chan and Webster repeated their Cochrane review in 2013 ^84^ but still found no evidence from randomised controlled trials, concluding that:

…with sustained concerns about the safety of the pathway implementation and the lack of available evidence on important patient and relative outcomes, recommendations for the use of end-of-life pathways in caring for the dying cannot be made ^84^ (p1).

## Transformation

The absence of ‘gold standard’ research evidence made it difficult to counter criticisms of the LCP that began to emerge beyond academia from 2009 onwards. Now more mainstream print media and the emerging power of social media were seen to combined effect as a range of ‘stakeholders’ colonised the LCP debate for a variety of political ends, ranging from ‘pro-life’ campaigners, to anti ‘Obama care’ activists in the USA. In between, the voices of people who had been bereaved also found a space. Many of these cited the LCP as a key factor in poor care, although others reported positive experiences of it.

### A gathering ‘storm’

Thunder clouds associated with the LCP appeared in the wider public domain as early as September 2009 with the publication of a short but highly critical letter in the Daily Telegraph ^85^ from six individuals, including: an emeritus professor of geriatric medicine, a consultant in palliative medicine, an anaesthetist, a lecturer and the Chairpersons of the Medical Ethics Alliance ^e^ and of ‘Choose Life ^f^’ (the latter both ‘pro-life’ organisations). The letter, which echoed concerns expressed in the clinical press by Craig a year earlier ^81, 82^, was published a week after the appearance of a report from the Patients Association ^86^ estimating that up to 1 million patients had received poor care in NHS hospitals, creating fertile ground for media and wider public interest. The authors of the letter noted the Patients Association report and immediately voiced their concerns that:

…a tick box approach to the management of death is causing a national crisis in care (and) …a nationwide wave of discontent … as family and friends witness the denial of food and fluids to patients ^85^.

Further, the authors claimed that many deaths ‘come about’ as a result of terminal sedation, picking up on a wide misreporting in the media of research by the sociologist Clive Seale. In a paper presenting a case study of media reporting of his research on end of life decisions, Seale analysed how media accounts acted as a conduit for introducing new considerations in the public debate about end of life care beyond the narrower confines of assisted dying, hitherto the field of most interest to journalists, albeit using familiar techniques of simplification and polarisation ^87^. Seale also notes that where something is considered to be ‘newsworthy’ (as was the case with the LCP ‘story’), a ‘feeding frenzy’ of ‘pack journalism’ can develop amongst the media ^87^ (p2).

In the case of the LCP, a health correspondent in the Daily Telegraph quickly picked up on the significance of the story and subsequently ran a series of short reports, the first of which had the memorable title: ‘Sentenced to death on the NHS’ ^88^. It was sufficient to obscure the inclusion in the article of observations from a range of agencies in support of the LCP, including Marie Curie and the Department of Health. Other pieces quickly followed, usually taking the familiar form of personal stories - the first of these from a woman who claimed her father had been wrongly placed on the LCP ^89^. A review of the correspondence in the Daily Telegraph undertaken subsequently by Mackintosh (2015) calculates that there were 431 individual comments in response to the original letter and the reports that followed ^90^.

As Seale ^87^ notes, the series of reports in the Daily Telegraph about the LCP, and especially the claim that sedation practices were causative of death, were picked up not only in the UK press but also in the USA by Lyndon Larouche, an American political activist who was opposed to what was widely known as ‘Obama Care’ (the Affordable Care Act):

On Sept. 3, Britain’s Daily Telegraph published a lead article featuring a Letter to the Editor from six prominent British doctors and health-care professionals, charging that large numbers of patients in the U.K. are being “sentenced to death,” by means of involuntary euthanasia. The numbers were stunning: According to a report from a researcher at Bart’s and the London School of Medicine and Dentistry, one out of six people who died in the United Kingdom in 2007–08, died of continuous deep sedation, the mode of euthanasia which the doctors describe. As we present the evidence, you will see precisely what the Obama Administration has in store for the United States in its full Nazi form. Executive Intelligence Review (web-based). Sep 11th, 2009. www.larouchepub.com (published in Seale, ^87^ p5-6)

In 2012 there was another flurry of reports and letters in the Daily Telegraph following a presentation on the 18th June 2012: “Is it possible to make a diagnosis of impending death? The scientific evidence” by Prof Patrick Pullicino (Prof of Neurosciences, University of Kent) at a conference of the Medical Ethics Alliance ^g; h^, in which he was highly critical of the LCP. Subsequently published in the Catholic Medical Quarterly ^91^, his paper saw the LCP as a form of institutionalised euthanasia ^i^. The Daily Mail quickly picked ups on Pullicino’s claims, and subsequently began a long running campaign against the LCP in articles with titles such as: ‘Care? No this is a pathway to killing people that doctors deem worthless’ ^92^.

Capitalising on the attention gained from reports of the talk by Pullicino, the authors of the 2009 letter to the Daily Telegraph, wrote again July 8th 2012, calling the LCP a ‘Deadly one way street’ ^93^. Soon afterwards, a letter to the Telegraph signed by over 1,000 working doctors, nurses and carers took the opposing view and expressed support for the LCP as a dignified way to die ^94^. The issues raised in the various print medium reports and letters also surfaced in social media.

### A space opens in social media for the bereaved to give voice

The media furore opened up a space within which the complaints of bereaved people, rather than health professionals, about end of life care began to be noticed. The essence of these centred on awareness of their relative’s dying, lack of involvement in decision-making and poor quality of communication. The use at that time of comparatively new social media such as Facebook and Twitter enabled what might be seen as an essentially ‘grass roots’ movement to come to wider attention, and provided a means of networking between individuals and organisations. This in turn furnished the mainstream media with more material, including reports or ‘think pieces’ published in the Guardian Society ^95^, the Daily Mail ^92^ and most other UK newspapers at regular intervals between 2010–2012. Most were critical and used extreme language. For example, the Daily Mail used the term ‘Liverpool Killing Pathway’ and went on to erroneously claim that the LCP was implicated in the deaths of young babies and children: the latter report was the subject of a Press Complaints Inquiry and was reported in the BMJ ^96^. Perhaps reflecting either an inability to gain newspaper publication or a fear of being misreported, most commentators seeking to lend support to the LCP did so in the traditional medical and health care press, although many were also active on Twitter.

Some correspondents on social media apparently sought not only to air their complaints but also to ‘bring down’ the LCP. For example, one Twitter hashtag @NHSNaziHunters ^j^ echoed the language of the anti-government activist Lyndon Larouche reported earlier. As the wide ranging media activity peaked, it culminated in a petition by the organisation Change.org for a national enquiry, which took the form of an open letter to the Health Secretary of the United Kingdom government ^k^.

### A rising sense of risk

Controversy and criticism continued unabated in spite of efforts to lend support to the LCP by a range of organisations across the sector ^l^, and reiteration in a variety of policy and practice documents that the LCP:

…requires senior clinical decision making, communication, a management plan and regular reassessment. It is not a treatment in itself but a framework for managing treatment. It aims to support, but does not replace, clinical judgment. Communication, care and compassion must come from all the healthcare workers caring for an individual and their family ^97^ (p17).

In 2012 a report that half of all NHS trusts had received, or were due to receive, financial awards for demonstrating use of the pathway under a system known as ‘Commissioning for Quality and Innovation’ (CQUIN), was cited in the Daily Telegraph as evidence of doubtful use of the LCP, and claiming undue financial and bureaucratic influence of individual clinical practice ^98^.

In January 2013, two stories were run in the Daily Telegraph reporting that a national enquiry into the LCP has been commissioned by the Norman Lamb, MP, Minister of State for Care Support. The first cited an interview with Dr Bee Wee (then president of the Association for Palliative Medicine):

…some hospitals appeared to be treating the pathway as just another “thing to be done” rather than something to be handled with extreme care. She added that the cases which have come to light suggested that “packaging up” principles used in hospices for hospitals had caused difficulties. “There’s a very big difference in the culture of hospitals,” she said. “So the environment and the attention to training and support ongoing is an important point” ^99^.

In the same article, Lamb, apparently capitulating to the growing storm, was reported as saying:

I have committed to appoint an independent chair to review how end of life care is working and oversee the reviews into the LCP. …This review will also consider the value of locally set incentives, and whether they are leading to bad decisions or practice ^99^.

As a national independent review panel was convened, key elements in the controversy were distilled into the content of a Channel 4 television programme in February 2013 called ‘Death on the Wards’, in the investigative series ‘Dispatches’. This broadcast sought to establish the truth or otherwise about accusations in the media about the LCP. A month later a survey was published of 647 UK hospital doctors, jointly conducted by the BMJ and Channel 4 ^100^, which found that critical reports of the LCP were negatively affecting its use. Many clinicians responding to the survey expressed concerns about distress caused to relatives of dying people by ‘scaremongering’ in the press about the LCP. Many were also anxious about how to respond. One respondent wrote this:

Negative press regarding LCP [the pathway] has caused additional distress for relatives at an already distressing time when their loved one is dying. This has caused a dilemma in judging if discussing the LCP will cause more distress than the benefit of being on the LCP for coordination of care in the dying phase ^100^ (p18).

The survey enquired into doctors’ views about the accusation in the press that the LCP was used to ‘save money’ ^101^. The vast majority of respondents (98%) did not think that resource considerations played a part in decisions to use the LCP, although most (58%) disagreed with the principle of using financial incentives provided to NHS Trusts for adoption of the LCP and other similar initiatives ^100^.

## The National Independent Review and its recommendations

The national independent review panel was chaired by Baroness Julia Neuberger and was given the brief of examining in detail the use and experience of the LCP in England and reporting its findings independently of the Government and the NHS ^m^ ^11^. The review panel comprised ten individuals including care campaigners, a journalist, hospice and hospital leaders, a senior doctor and nurse, and an academic ^n^. It met five times between February and June 2013 to consider a range of evidence, including: written submissions from health care professionals (n=91), members of the public (n=483), professional bodies and other organisations (n=36); a rapid review of research evidence about the key components of integrated pathways in the dying phase of end of life care ^o^ ^20^; a snapshot review of hospital complaints and the results of the survey of health care professionals referred to earlier ^100^. In addition, the panel met members of the public at four sessions. The review panel concluded from the evidence received that:

…where the LCP is used properly patients die a peaceful and dignified death. But the review panel is also convinced, from what it has heard and read, that implementation of the LCP is not infrequently associated with poor care ^11^ (p7).

Overall, the panel made 44 recommendations, organised under 25 themes ^p^, of which the very large majority provide an agenda to improve the quality of care for dying people and their relatives that extends far beyond the scope and remit of the LCP as originally conceived, and which at the time of writing this paper were still being developed, discussed and disseminated. As well as proposals to cease reference to the LCP because of confusion about the term ‘pathway’, the recommendations addressed deficiencies in: documentation; prognostication and diagnosis of dying; communication and clinical decision-making at the bedside (especially in relation to nursing practice surrounding clinically assisted and oral hydration and nutrition, provision of pain relief and sedatives), consent; care planning; cardio-pulmonary resuscitation and other ethical issues. Wider aspects were also considered, including the quality of the environment of care and staff resources and equipment. Likewise, a proposal was made for a system-wide strategic approach to be adopted to improve care of the dying, building on the foundations of the End of Life Strategy published five years earlier in 2008 ^10^, with end of life care incorporated into hospital inspections.

In the press release ^q^ accompanying the review, Baroness Neuberger stated that:

All the major players in the health and care system, including the Government, need to do their part in reforming care for the dying, so that people everywhere can be sure they will be treated with respect and compassion, supported to die a peaceful, dignified death.

The panel concluded that use of the LCP should be phased out by July 2014, with the intention that it be replaced by a personalised ‘end of life care plan’ backed up by good practice guidance specific to disease groups. Interim guidance to this effect was published for doctors and nurses on 16 ^th^ July 2013 by NHS England ^102^. On 15 ^th^ August, and after the publication of the Neuberger report, BBC Radio 4 broadcast a 30 minute programme assessing the rise and demise of the LCP and its future implications. There were contributions from family members of patients who had been put on the LCP, as well as from palliative care specialists, including Professor Ellershaw ^103^.

## Aftermath

### New guidance develops

The independent review panel primarily directed its recommendations about the LCP to a range of national organisations with statutory or regulatory roles in health care (for example: NHS England, Care Quality Commission, Department of Health, NICE; General Medical Council). In England, quite quickly after the publication of the independent review, representatives from these organisations together those from a range of others, including charities, formed a coalition of 21 known as the ‘Leadership alliance for the care of dying people’ (LACDP). The stated purpose of the alliance was ‘…to take collective action to secure improvements in the consistency of care given in England to everyone in the last few hours or days of life and their families’ ^104^ para 3, with the following objectives: first, to support all those involved in the care of dying people to respond to the findings of the review; and second, to be the focal point for the system’s response to the findings and recommendations of the LCP review. The LACDP’s approach was to develop five ‘priorities of care for the dying person’ (see Box 3).

**Box 3.** Priorities for care for the dying person published by the Leadership Alliance for the Care of the Dying Person in 2014 ^103^

The Priorities for Care are that, when it is thought that a person may die within the next few days or hours: 1. This possibility is recognised and communicated clearly, decisions made and actions taken in accordance with the person’s needs and wishes, and these are regularly reviewed and decisions revised accordingly. 2. Sensitive communication takes place between staff and the dying person, and those identified as important to them. 3. The dying person, and those identified as important to them, are involved in decisions about treatment and care to the extent that the dying person wants. 4. The needs of families and others identified as important to the dying person are actively explored, respected and met as far as possible. 5. An individual plan of care, which includes food and drink, symptom control and psychological, social and spiritual support, is agreed, co-ordinated and delivered with compassion.

The priorities were worded to direct attention to the importance of individualised assessment and care planning, and away from the standardised approach that the review panel found had sometimes been adopted in the use of the LCP. The alliance emphasised that ‘…where change is needed, it is in the practice of particular local organisations and staff’ ^104^ (p7), with the role of national organisations to: ‘…require, encourage and support that change’ ^104^ (p7). In addition, Alliance members agreed and published a ‘commitment statement’, setting out their individual and collective approaches to improved care in the last hours and days of life. The following year an editorial published in the British Medical Bulletin by a leading palliative care doctor, Dr. Nigel Sykes, communicated the step change in shared responsibility for end of life care that he believed the Alliance’s report introduced:

…. doing the right clinical thing is no longer the sole responsibility of care providers. In addition, the role of contracting and resources is recognized through an explicit expectation that Commissioners of care will share the responsibility for effective end-of-life care, while previous training deficiencies are acknowledged through placing this responsibility also with Commissioners of education and training as well as the medical and nursing Royal Colleges ^105^ (p45).

A parallel process in Scotland, co-ordinated by the Living and Dying Well National Advisory Group ^r^, resulted in the publication in December 2014 by NHS Scotland of a guide and set of four principles entitled ‘Caring for People in the Last Days and Hours of Life’ ^106^. It was designed to complement the broader ‘Scottish Palliative Care Guidelines’ ^107^ published in November 2014 by a multi-disciplinary group of professionals, public partners and in collaboration with the Scottish Partnership for Palliative Care and Health Improvement Scotland. In Wales, where an an integrated care pathway for the last days of life based on the LCP had been widely used, the All Wales End of Life Care Programme developed new guidance ‘Care Decisions for Last days of Life’ to incorporate the recommendations of the Neuberger and LACDP reports ^108^.

The work of the LACDP in England was followed by the development and publication by NHS England of ‘Actions for End of Life Care 2014–2016’ ^109^, aimed at the care of adults and children, and the publication (in December 2015) by the National Institute of Clinical Excellence of a guideline on care of dying adults in the last 2 to 3 days of life ^110^. The target audiences were health and social care professionals, commissioners and providers of care, as well as patients and their families/carers. The contextual overview of this document neatly summarises the largely agreed view on the LCP that had by now been reached:

Although the LCP was designed to bring values of good end of life care from the hospice movement to mainstream hospitals and elsewhere, it met with increasing criticism from the public, health care professionals and the media. There were 3 main areas of concern:
  • recognising that a person was dying was not always supported by an experienced clinician and not reliably reviewed, even if the person may have had the potential to improve
  • the dying person may have been unduly sedated as a result of injudiciously prescribed symptom control medicines
  • the perception that hydration and some essential medicines may have been withheld or withdrawn, resulting in a negative effect on the dying person.
These were not necessarily a direct consequence of following the LCP, but often happened because of poor or indiscriminate implementation or a lack of staff training and supervision ^110^ (p5).

As we will see below, the review of the LCP and the subsequent questions of implementation and quality raised by NICE were much debated in the health care press, especially in the academic research journals related to palliative care.

### Published commentaries and editorials: key themes

Following its withdrawal, a large number of editorials and comment pieces were published on the theme of ‘what went wrong with the LCP’? Here three major themes were in evidence: 1) the LCP as a iconic example of what happens when ‘gold standard’ evidence from randomised controlled trials is not gathered before adoption of a complex intervention; 2) the LCP as a failure of implementation rather than any fundamental problem in its formulation; and 3) the LCP as something misunderstood and abandoned without due cause.

Straddling the first two of these is an editorial in the *BMJ* by Sleeman and Collis about the LCP ^111^:

Was this a failure of the paperwork or of its implementation? If implementation and training are key, would investment in these areas—rather than developing guidelines from scratch—be a more efficient use of resources? The lack of strong evidence of the LCP’s benefits undermines this argument. However, the converse is also true: the absence of prospective evidence of harm should caution us against the assumption that simply withdrawing the LCP will improve end of life care…. Ultimately, the decision to phase out the LCP was made on the basis of little more than an accumulation of anecdotal evidence. Without independent prospective evidence from controlled trials, the LCP became unusable. This should serve to warn us of the dangers of the national implementation of tools that are not properly evidence based ^111^ (p7).

A year later and in similar voice, an editorial in the Lancet ^12^ by Currow and Abernethy accompanying the publication ^112^ of the first RCT of the LCP, asserts:

…the LCP was taken up by bureaucrats who did not understand the implications of widespread implementation of an initiative for which the net effects were poorly defined … As demonstrated by the results of Costantini and colleagues’ study, a government, when introducing such initiatives, should properly assess them in rigorous trials of health services, preferably randomised; if this cannot be achieved then a formal prospective assessment of new interventions as they are implemented must be the minimum standard. Either assessment should be done in a health-care environment where new interventions are thoughtfully introduced, corresponding data are routinely gathered for the interventions, and analyses inform understanding of the net benefit and opportunities for iterative enhancement—namely, a learning health system framework, as described by the US Institute of Medicine ^12^ (p192).

The multicentre cluster randomized trial ^112^ to which Currow and Abernethy’s editorial ^12^ refers was carried out in Italian hospitals across 16 general medicine wards with at least 25 deaths from cancer annually, and involved 308 patients who died from cancer and their families. Although some commentators have argued that the study was underpowered ^15, 111^ and subject to bias for methodological reasons ^15^, it was well designed and rigourous. It found no significant difference in overall rating of quality of care between patients who died in wards in which the LCP had been implemented when compared with those in which it had not, although improvements were observed in two out of 9 secondary outcomes (respect and control of breathlessness). No differences in survival or medication use were observed. The ‘take home’ message from the cluster trial was that any benefit derived from the LCP depends on the quality of its implementation. Since then, a cluster randomized trial of the ‘Care Programme in the Last Days of Life’ (CAREFul) has been conducted in the Netherlands, based on the LCP and taking account of its critique ^113^, finding that the progamme improved nurse assessed comfort but not satisfaction with care among relatives. The authors conclude that further qualitative research is required to gain an understanding of this apparent discrepancy and recommend ‘controlled implementation’ with due regard for ongoing training of clinicians especially in communication skills ^113^ (p132).

A similar theme of implementation is picked up by the American authors Billings and Block in a review in the *Journal of Palliative Medicine* ^114^ which noted that:

…the tragedy of the LCP lies in the gap between the apparent value of its guidance for clinical care in the last few days of life and its performance in daily practice ^114^ (p1493).

These commentators also draw four wider conclusions: first, that the Neuberger report itself may be criticized for not being a scientific study of the LCP and for relying too much on anecdote; second, that the LCP story has revealed serious deficiencies in the perception of palliative care in the UK; third, that it reveals serious deficiencies in care in NHS hospitals which go far beyond the scope of the LCP; and fourth, that engagement of anti-euthanasia groups clearly impacted on the reputation of the LCP.

Nor did political and media interest in the LCP disappear. When in late 2015 evidence was given to a Health Select Committee of MPs, confirming that thinly disguised versions of the LCP were in use in some settings despite the Neuberger recommendations, the Daily Mail once again returned to the issue, this time condemning the ‘arrogance’ of doctors who were flying in the face of best practice ^115^. Prominent figures in the palliative care world, including Professor Sam Ahmedzai, who chaired the NICE guidelines committee, were equally vocal in the condemnation. 

## Discussion

Following Carlile’s ^28^ description of boundary object implementation as a process involving *transfer, translation and transformation*, we have shown how the LCP quickly assumed national prominence as a key means to deliver the goals of a National End of Life Care Strategy ^10^. On entering the phase of transformation, it became a site of expression and a signifier of difference around a range of domains that extended far beyond its original conceptualization as a guide for clinical staff caring for people at the end of life. It was some five years into the national roll-out of the LCP that a sense of public scandal began to break. As Butler and Drakeford (2005) show in their analysis of scandals, it was not unusual that the public furore began with a letter to a broadsheet newspaper. Research on scandals in health and social welfare suggests that some indeed can be iconic in character, leading to a fundamental shift in public awareness and thinking. This can be positive for the wider issue at hand (care in the last days of life, care of the dying) if negative for the specific intervention in question (in this case the LCP). Scandals are socially constructed. Similar phenomena may elicit different responses. As the scandal spiralled outwards the LCP came to signify ‘end of life care’ in its totality. Some of the arguments expressed were of much wider import and went well beyond the specific goals of the LCP. These included: 1) clinical resourcing issues, with the LCP story exposing some fundamental fault lines in an over extended and under resourced NHS; 2) research, with the LCP becoming a platform for debates about the nature of the ‘evidence’ required to underpin innovations in health care generally and palliative care specifically; 3) the moral and ethical domain, with the LCP being used as a conduit of discord around what constitutes ‘good’ or ‘bad’ practice in care of the dying; and 4) the public and media domain, where the voice of bereaved people found expression, and the power of emergent social media in creating new knowledge and understandings became evident.

The transfer and translation of the LCP took placed in the context of a widespread and uncritical enthusiasm for integrated care pathways that was gathering momentum in many modern health systems in the early 2000s. The LCP caught the wave of this, but in her description of integrated pathways as classical examples of boundary objects, Allen ^27^ (p355) notes that while they have a ‘strong cohesive power’ to appeal to a range of stakeholder group, their breadth of appeal also disguises tensions between different agendas and frames of reference. To this extent, the development and evolution of boundary objects such as the LCP must always be understood as a political process, with the resultant tools functioning as ‘embodied practices for routing patients through the system’ ^17^ not neutral mechanisms ^19^. As we have illustrated, before the LCP came to the awareness of the wider media, it encapsulated an ambiguity shared with all other ‘pathways’, between standardisation and person centred care ^16^. This ultimately undermined its core purpose of improving decisions for each individual patient’s care in spite of efforts by its promoters to build in safeguards against poor practice and the publication of version 12 in response to criticism. The problem of ambiguity was compounded by an inadequate focus in the wider NHS on clinicians’ underlying knowledge in palliative care principles and ethical approaches to end of life decision-making ^11^. In a cogent discussion of this latter issue, an Australian palliative medicine specialist, Mackintosh, has observed:

…it was not so much about what was said in the LCP documentation as what was not said and brings to light the difficulties of end of life decision-making. Ticking two out of four boxes about symptoms that were not specific to the dying patient now seems a rather naive approach; diagnosing dying can be a complex process filled with uncertainties ... specialist palliative care teams make their decisions very carefully following all the safeguards contained within the LCP. Much of what is implicit in the practice of specialist palliative care was never made explicit; the V12 algorithm came too late ... the LCP has been accused of adopting a 'one size fits all' approach. However, this was not the case as a careful examination of the documentation will reveal. Instead the LCP was interpreted as being a 'one size fits all' tool to practitioners without the required skills to read between the lines ^90^ (p651).

The LCP was understood by its designers to be a complex intervention and, as we have seen, for its implementation they recommended ongoing training and support of clinicians at an organisational level to be provided by expert facilitators. As the LCP was rolled out at scale, this aspect was weakened and the LCP began to be represented as a stand-alone document ^s^, rather than a broad approach to care in which the documentation was merely one aspect. Lack of training, together with ambiguities and misunderstandings surrounding the LCP, were combined with rapidly increasing pressures on the workforce. Indeed the Review Panel observed ‘constant pressures on staff and that some find the workload unmanageable’ ^11^ (para 2.25), with hospitals in England running at 90% capacity rather than the 80% recommended for safety. The LCP story thereby highlighted an unpalatable truth of much broader significance: when healthcare workers are exhausted and overstretched, quality of care, communication and decision-making are compromised ^116^. As other commentators have concluded: ‘…services that provide poor quality general care will undoubtedly provide very poor end-of-life care ^117^ (p510).

As the LCP story unfolded, it became a conduit for debate about the proper epistemic foundations of innovation in health care (quality improvement versus evidence based medicine). The LCP was not originally underpinned by research evidence from RCTs, but was far from alone in this as an intervention in palliative care or in health care more generally. For example, there was no LCP-type reaction to the ‘Gold Standards Framework’ ^118^ or ‘Preferred Priorities of Care’ ^119^, which were similarly high profile boundary objects promoted by the National End of Life Care Programme, each lacking supporting evidence from RCTs. Moreover, Mackintosh ^90^ has argued that the calls for better evidence in the LCP debate are misplaced ‘….since what is being measured is not the performance of the tool but the performance of the user’ (p651) . The RCT evidence that has since emerged from Italy ^120^ and from the Netherlands ^113^ powerfully draws attention to this point.

 An essential characteristic of boundary objects described by Star is their interpretive flexibility ^121^. Where over standardization occurs this works against the reflective use and local tailoring that enables boundary objects to be used effectively. In her prescient case study of the ways in which integrated care pathways develop, Allen ^27^ concludes that diversity rather than standardization should be encouraged, thereby ensuring their utility for different purposes and contexts:

They should not be treated as pre-fabricated tools that supposedly can be easily transferred from one context to another. Furthermore, it is not sufficient to introduce a boundary object and wait for it to do its magic. The local process of making, introducing and using care pathways is crucial for making them work as boundary objects and continuous work is needed to sustain them ^27^ (p 648).

It is well known that boundary objects need to be accompanied by the involvement of ‘knowledge brokers’ to enable translation into practice ^122^. This was something recommended by the Marie Curie Palliative Care Team in their recommendations for implementation (albeit using different terminology) ^123^, and highlighted in the conclusions drawn from the results of the randomised controlled trial of the LCP in Italy ^112^. Knowledge brokers were also found to be critical to the success of the wider National End of Life Care Programme in an evaluation of its first phase ^48^. Wenger describes such brokerage as a ‘… process of translation, co-ordination and alignment between perspectives. It also requires the ability to link practices by facilitating transactions between them’ ^124^ (p38). Following its withdrawal in England, a study in the Netherlands ^125^ on barriers and facilitators to implementation of the LCP reported on the importance of skilled and ongoing facilitation, as well as ongoing training for the continuous development of competence in palliative and end of life care in the workforce. Similarly, a review by McConnell *et al.* ^126^ examining what hindered the implementation of the LCP showed that successful interventions are configured to address and influence the understandings of staff about end of life care and to increase their motivation and self–efficacy, and highlighted the support of senior managers as essential, both to release necessary resources and to enable culture change in organisations.

Integrated care pathways are simultaneously work flow models and records of care ^27^. They are both management and clinical tools. As Allen ^27^ demonstrates, they also appeal to two distinct but related logics within the health care system. Pathways are about evidence based practice as well as quality improvement. If the former suggests scientific knowledge that is slow to accumulate and problematic to implement, the latter promotes local initiatives, quick implementation, adaptation and rapid feedback into practice. The LCP embodied these tensions. The formal evidence from a randomised control trial that brought its efficacy into question did not come until after major concerns had led to recommendations for its withdrawal. At the local level however, LCP was subject to annual scrutiny by a multi-disciplinary steering group that included carer representation and over time it moved through a series of revisions and numbered versions. Its ‘roll out’ was therefore the product of quality improvement enthusiasms at local level which were quickly scaled up. In this LCP can be seen as a positive ‘boundary object’ with the potential to enrol clinicians, managers, service users and indeed wider publics in the common aim of improving care of the dying. When this broke down however, LCP was transformed into a negative boundary object, which served to highlight not shared enrolment, but rather fundamental conflicts of view. The LCP is not alone in this. As Allen’s studies show ^22, 27^, other pathways have undergone this transition – and have been quietly abandoned in the process before a crisis occurred. The LCP however was unusual in becoming high profile. It was not obscured within the complexities of daily clinical practice, but rather was championed as the diffusion of hospice principles to meet the needs of the majority rather than the few. It was heralded as a transformative intervention to improve system-wide terminal care. Although its protagonists were always careful to emphasise that the LCP was only as good as the skills of those using it, the LCP, as it quickly rolled out, ran ahead of these skills ^126, 127^.

Hendy and Barlow ^128^ have shown that organisational champions can be highly effective in the initial phases of health system innovation, when change is contained within distinct sub-sets of practice. But they caution against change being positioned in the hands of too few individuals, which may prove detrimental to wider implementation. The LCP may have suffered from this problem. It might also be argued that the relevant champions lacked foresight. In environmental areas it has become routine to practice ‘responsible innovation’ ^129^, acknowledging that innovation can raise questions and dilemmas, is often ambiguous in its purposes and motivations and can be unpredictable in its effects, beneficial or otherwise. The approach operates on four principles of: 1) anticipating impacts, intended or not 2) reflecting on potential motivations, uncertainties, framings, dilemmas and transformations that might ensue 3) engaging in dialogue and debate about these issues in an inclusive way 4) acting to influence the direction and trajectory of the innovation process. Such principles could map fairly easily onto the LCP narrative, but were largely absent in practice. In a related way, Greenhalgh and colleagues ^130^ have published a framework for understanding abandonment and challenges to the scale up of health and care technologies. They show that innovations that fail to address complexity across 7 specific domains are unlikely to be sustainable: (i) nature of illness and co-morbidities; ii) material features of the technology and knowledge needed to use it; iii) values (including efficacy, safety and cost-effectiveness); iv) adopters (including staff roles and practices); v) organisation (organisational readiness to adopt and implement); vi) wider system (consideration of professional and stakeholder perspectives) and vii) scope for embedding and adaptation over time. As our account shows, several of these factors are relevant to the rise and fall of the LCP.

The sequence of events surrounding the LCP brought to an end the era of ‘unconditional regard’ ^4^ for palliative care. No longer could it be assumed that palliative care was universally welcomed. The LCP exposed ‘sites of difference’ upon which the goals of care and methods of care at the end of life were opened up to wide scrutiny. The ramifications seem not yet to have been fully acknowledged by those involved.

## Conclusion

We set out to answer three sets of questions: 1) why and how did the LCP come to prominence as a vehicle of policy and practice 2) what factors contributed to its demise? 3) what immediate implications and lessons resulted from its withdrawal?

The LCP emerged in response to a clear need for ‘scaled up’ approaches to care interventions at the end of life. The case in the late 1990s was compelling. The available provision of palliative care through hospices and specialist palliative care units in hospitals was clearly incommensurate with the prevailing level of need for such services and the number of people who could benefit from them. This was therefore addressed through the development of a mechanism that fitted with then current enthusiasms for integrated care pathways and which could bring some of the essentials of palliative care practice to a wide range of beneficiaries. The initial success of the approach, what might be called the period of the LCP as successful boundary object, can be measured by the traction that it gained in its roll out and in the early results from local and service improvement studies. This was based on successful involvement from a number of stakeholders – across health care disciplines, hospital management and service users. Such an approach resonated strongly at that time with a predisposing climate of policy interest in the provision of multi-disciplinary end of life care and a strategic commitment to support the transfer of successes seen in the British hospice context, into the wider health and social care system.

The factors that contributed to the demise of the LCP in turn relate to how it then broke down as a boundary object. The LCP needed constant cultivation by knowledge brokers if it was to hold together and sustain the engagement of disparate stakeholders. Where this faltered, the default position was one of mechanistic and potentially insensitive implementation of the LCP as a protocol or checklist. It then became the site of conflicts provoked by ideologically inspired interest groups, both within and beyond the healthcare environment, which fostered a previously unseen critique of certain assumptions within the palliative care paradigm. This found its correlates in the media and wider expressions of public concern. As these grew, the LCP became the bearer of responsibility for all aspects of NHS end of life care, well beyond its original remit, goals and aspirations.

Meanwhile, palliative care experts were slow to respond to these concerns, or to develop evidence about the LCP in relation to which they could be judged. Instead their contributions only emerged with any significance in the aftermath of withdrawal when some hastened to publish their thoughts. The contributions polarised between those who had ‘always’ had reservations about the LCP and those who saw its demise as a matter of regret and who in turn challenged the critics to present a better alternative. The five ‘priorities of care for the dying person’ developed by the LACDP and the subsequent NICE guidance, with its flimsy evidence base, were long on values and aspirations, but short on a practical course of action. They replaced one set of deficiencies with another.

On the available evidence and within the limits of our chosen method here, we judge that the LCP boundary object was well conceived, but not matched by sufficient subtlety and foresight in its implementation and dissemination. The dramatic demise, when it came, resulted from a combination of media-fuelled public criticism and long-delayed professional judgement, hitherto never experienced in the developing field of palliative and end of life care. The most important lesson that can be learned however is not about the dangers of scaling up clinical interventions that lack an ‘evidence base’. Rather it is about the need for greater assessment of the wider risks involved and more careful consideration of the unintended consequences that might result from a given course of action – especially in the politically and morally charged arena of end of life care interventions.

## Data availability

All data underlying the results are available as part of the article and no additional source data are required. The paper is based on an analysis of documents in the public domain.

## Notes

^a^The audits were carried out by the Marie Curie Palliative Care Institute Liverpool (MCPCIL) in collaboration with the Royal College of Physicians (RCP) London, supported by Marie Curie Cancer Care and the National End of Life Care Programme at the Department of Health. In round 2, 155 hospitals from 114 Acute Hospital Trusts participated in the audit and submitted 3893 patient data sets (Source: http://www.mcpcil.org.uk/media/16531/generic_ncdah_2nd_round_final_report[1].pdf Accessed August 31st, 2017).

^b^Matthews *et al.* report introduction of the LCP into paediatric care

^c^Anecdotally, one of the authors (Seymour) recalls attending a conference hosted by the Marie Curie Institute of Palliative Care which was disrupted by a protest about the LCP from members of the Medical Ethics Alliance.

^d^Qualitative Studies =4; Health Professional and/or Carer Perceptions= 6; Pre- and/or Post-Pathway Audits=10; Retrospective Symptom Management=3; Benchmarking: 5

^e^The Medical Ethics Alliance is closely associated with Catholic ethics’ agencies. Catholic doctors and hospital chaplains played a key role in the original and development of the LCP but others were centrally involved in the critique.

^f^‘Choose Life’ is a strap line used by The Christian Institute, www.christian.org.uk which campaigns on a wide variety of social and ethical issues (Accessed July 12th 2017). 

^g^The programme is available here http://www.medethics-alliance.org/index.php/2014-11-02-22-25-35/events/87-natural-death-do-we-need-a-pathway (Accessed July 12th, 2017)

^h^There is also material on the website of the Medical Ethics Alliance refuting a supportive statement on the LCP published by the National End of Life Care Programme http://www.medethics-alliance.org/index.php/2014-11-02-22-25-35/press-releases/92-commentary-on-the-statement-supporting-the-liverpool-care-pathway (Accessed July 12th 2017)

^i^This is the subject of a robust response in a blog https://illusionsofautonomy.wordpress.com/2012/11/30/a-critique-of-pure-unreason-response-to-the-d/ (Accessed July 12th 2017) by Dr Peter Berry posted on the 30th November 2012, Berry writes this:

My overriding objection to this paper centres on the use of the word euthanasia. It is suggested that widespread use of the LCP equates to institutionalised euthanasia, and implicit in this is an accusation that individual practitioners have killed their patients. To read this, as a doctor who has used the LCP, is very difficult. The accusation is made in the conclusion without any supporting evidence. The ‘evidence’ that is reviewed in the paper does not touch upon intentional killing. If we are regularly making inaccurate predictions (or prognoses), that is of course unacceptable and must be addressed, but the term euthanasia suggests that we are intentionally killing our patients. There is absolutely no evidence for this. This paper, and the thoughts behind it, sparked a huge controversy over end of life care in this country. I think it is methodologically weak and structurally flawed. I think it contains baseless conclusions, and is excessively liberal with emotive, hurtful accusations of intentional killing.

^j^ https://twitter.com/nhsnazihunters?lang=en-gb Accessed May 10th 2017

^k^ https://www.change.org/p/the-health-secretary-rt-hon-jeremy-hunt-avoidable-deaths-in-our-hospitals-a-public-enquiry-into-the-end-of-life-care?recruiter=110192815&utm_source=share_petition&utm_medium=twitter&utm_campaign=share_twitter_responsive Accessed 10th May 2017

^l^ http://cno.dh.gov.uk/2012/11/20/media-round-up-end-of-life-care-and-the-liverpool-care-pathway/ (Accessed July 12th 2017)

^m^Detailed terms of reference can be found on page 50 of the report.

^n^Baroness Neuberger (chair); David Aaronovitch (The Times); Tony Bonser (fund raiser Macmillan Cancer Support). Denise Charlesworth-Smith (National campaigner LCP after her father’s death), Dr Dennis Cox (RCGP); Lord Charles Guthrie (hospice chairman), Lord Khalid Hameed, Chairman Alpha Hospital group and London International Hospital), Professor Lord Harries of Pentregarth (Former Bishop of Oxford), Professor Emily Jackson (Professor of Law, LSE), Sarah Waller (Former trust nurse chief and director of human resources, lead of the King’s Fund Enhancing the Healing Environment programme).

^o^The lead author, Seymour, was one of the authors of this report.

^p^The 25 themes were: terminology; evidence base; falsification of LCP documentation; diagnosis of dying and prognostic tools; diagnosis of dying and communicating uncertainty; guidance on diagnosis of dying; good practice for nurses on decision-making; decisions to initiate an end of life care plan out of hours; training in shared decision-making; nutrition and hydration; sedation and pain relief; financial incentives; accountability; documenting an end of life care plan; independent advocacy; availability of palliative care support; guidance for nurses in end of life care; education in care of the dying; guidance; end of life care plans; a system wide, strategic approach to improving care of the dying; hospital inspections; thematic review of end of life care; and commissioning and mandate to NHS England.

^q^ https://www.gov.uk/government/uploads/system/uploads/attachment_data/file/212452/Press_notice_-_Liverpool_Care_Pathway.pdf Accessed August 31st, 2017

^r^The Scottish Partnership for Palliative Care describes the role of the advisory group here: https://www.palliativecarescotland.org.uk/content/living_dying_well/ (Accessed July 12th 2017) 

^s^For example, the Independent Review of the LCP (paragraph 1.15) notes that the Department of Health’s End of Life Care Strategy used wording which contributed to this impression.

## Supplementary material

Supplemental Material 1: An example of LCP documentation (July 2012).

Source: http://www.wihb.scot.nhs.uk/edocman/Liverpool-Care-Pathway.pdf (accessed July 11, 2017)
