# Supplementary material for: The Liverpool Care Pathway for the Dying Patient: a critical analysis of its rise, demise and legacy in England
Source: Wellcome Open Res. 2018 Apr 24;3:15. Originally published 2018 Feb 22. [Version 2] doi: 10.12688/wellcomeopenres.13940.2 (PMC5963294; doi:10.12688/wellcomeopenres.13940.2)
Supplement: Supplementary file 1 [file wellcomeopenres-3-15857-s0000.tgz › ce990d9d-4d29-417b-8786-5eb71d2c280d.pdf]

Name:... NHS no:... Date:...

Liverpool Care Pathway for the Dying Patient (LCP) supporting care in the last hours or days of life

Information sheet to be given to the relative or carer following a discussion regarding the plan of care.

The doctors and nurses will have explained to you that there has been a change in your relative or friend's condition. They believe that the person you care about is now dying and in the last hours or days of life.

The LCP is a document which supports the doctors and nurses to give the best quality of care. All care will be reviewed regularly.

You and your relative or friend will be involved in the discussion regarding the plan of care with the aim that you fully understand the reasons why decisions are being made. If your relative or friend's condition improves then the plan of care will be reviewed and changed. All decisions will be reviewed regularly. If after a discussion with the doctors and nurses you do not agree with any decisions you may want to ask for a second opinion.

Communication

There are information leaflets available for you as it is sometimes difficult to remember everything at this sad and challenging time. The doctors and nurses will ask you for your contact details, as keeping you updated is a priority.

Medication

Medicine that is not helpful at this time may be stopped and new medicines prescribed. Medicines for symptom control will only be given when needed, at the right time and just enough and no more than is needed to help the symptom.

Comfort

The doctors and nurses will not want to interrupt your time with your relative or friend. They will make sure that as far as possible any needs at this time are met. Please let them know if you feel those needs are not being met, for whatever reason.

You can support care in important ways such as spending time together, sharing memories and news of family and friends.

Information sheet to be given to the relative or carer continued:

Reduced need for food and drink

Loss of interest in and a reduced need for food and drink is part of the normal dying process. When a person stops eating & drinking it can be hard to accept even when we know they are dying. Your relative or friend will be supported to eat and drink for as long as possible. If they cannot take fluids by mouth, fluids given by a drip may be considered.

Fluids given by a drip will only be used where it is helpful and not harmful. This decision will be explained to your relative or friend if possible and to you.

Good mouth care is very important at this time. The nurses will explain to you how mouth care is given and may ask if you would like to help them give this care.

Caring well for your relative or friend is important to us. Please speak to the doctors or nurses if there are any questions that occur to you, no matter how insignificant you think they may be or how busy the staff may seem. This may all be very unfamiliar to you and we are here to explain, support and care.

We can be reached during daytimes at:……………………………………………………

Night time at:………………………………………………………………………………………

Other information or contact numbers (e.g. palliative care nurse / district nurse):
……………………………………………………………………………………………………
……………………………………………………………………………………………………
……………………………………………………………………………………………………
……………………………………………………………………………………………………
……………………………………………………………………………………………………

This space can be used for you to list any questions you may want to ask the doctors and nurses:
……………………………………………………………………………………………………
……………………………………………………………………………………………………
……………………………………………………………………………………………………
……………………………………………………………………………………………………
……………………………………………………………………………………………………..

© Marie Curie Palliative Care Institute Liverpool (MCPCIL)

Name:.. NHS no:.. Date:...

Liverpool Care Pathway for the Dying Patient (LCP)
supporting care in the last hours or days of life

Location: **(e.g. hospital, ward, care home etc.):...**

As with all clinical guidelines and pathways the LCP aims to support but does not replace clinical judgement

- The LCP generic document guides and enables healthcare professionals to focus on care in the last hours or days of life. This provides high quality care tailored to the patient's individual needs, when their death is expected.
- Using the LCP in any environment requires regular assessment and involves regular reflection, challenge, critical senior decision-making and clinical skill, in the best interest of the patient. A robust continuous learning and teaching programme must underpin the use of the LCP.
- The recognition and diagnosis of dying is always complex; irrespective of previous diagnosis or history. Uncertainty is an integral part of dying. There are occasions when a patient who is thought to be dying lives longer than expected and vice versa. Seek a second opinion or specialist palliative care support as needed.
- Changes in care at this complex, uncertain time are made in the best interest of the patient and relative or carer and needs to be reviewed regularly by the multidisciplinary team (MDT).
- Good comprehensive clear communication is pivotal and all decisions leading to a change in care delivery should be communicated to the patient where appropriate and to the relative or carer. The views of all concerned must be listened to and documented.
- If a goal on the LCP is not achieved this should be coded as a variance. This is not a negative process but demonstrates the individual nature of the patient's condition based on their particular needs, your clinical judgement and the needs of the relative or carer.
- The LCP does not preclude the use of clinically assisted nutrition or hydration or antibiotics. All clinical decisions must be made in the patient's best interest.
- A blanket policy of clinically assisted (artificial) nutrition or hydration, or of no clinically assisted (artificial) hydration, is ethically indefensible and in the case of patients lacking capacity prohibited under the Mental Capacity Act (2005).
- For the purpose of this LCP generic version 12 document - The term best interest includes medical, physical, emotional, social and spiritual and all other factors relevant to the patient's welfare.

The patient will be assessed regularly and a formal full MDT review must be undertaken every 3 days.

The responsibility for the use of the LCP generic document as part of a continuous quality improvement programme sits within the governance of an organisation and must be underpinned by a robust education and training programme.

References:

Ellershaw and Wilkinson Eds (2003) *Care of the dying*: *A pathway to excellence*. Oxford: Oxford University Press.
National Institute for Clinical Excellence (2004) Improving Supportive and Palliative Care for Adults with Cancer. London, NICE
MCPCIL (2009) National Care of the Dying Audit Hospitals Generic Report Round 2. www.mcpcil.org.uk

© Marie Curie Palliative Care Institute Liverpool (MCPCIL)

Name:.. NHS no:... Date:...

Algorithm – Decision making in: diagnosing dying & use of the LCP supporting care in the last hours or days of life

Assessment

Deterioration in the patient's condition suggests that the patient could be dying

Multidisciplinary team (MDT) assessment

- Is there a potentially reversible cause for the patient's condition e.g. exclude opioid toxicity, renal failure, hypercalcaemia, infection
- Could the patient be in the last hours or days of life?
- Is Specialist referral needed? e.g. specialist palliative care or a second opinion

Clinical Decision

Patient is **NOT** diagnosed as dying (in the last hours or days of life)

- Review the current plan of care
- Discussion with the patient and relative or carer to explain the new or revised plan of care

Patient is diagnosed as dying (in the last hours or days of life)

- Patient, relative or carer communication is focused on recognition & understanding that the patient is dying

Communication

- Discussion with the patient, relative or carer (IMCA as required) to explain the current plan of care & use of the LCP

Management

- The Liverpool Care Pathway for the Dying Patient (LCP) is commenced including ongoing regular assessments

Reassessment

A full multidisciplinary team (MDT) reassessment & review of the current plan of care should be triggered when 1 or more of the following apply:

- Improved conscious level, functional ability, oral intake, mobility, ability to perform self-care

and or

- Concerns expressed regarding management plan from either patient, relative or carer or team member

and or

- It is **3 days** since the last **full** multidisciplinary team (MDT) assessment

(→ return to Multidisciplinary team (MDT) assessment)

Always remember that the Specialist Palliative Care Team are there for advice and support, especially if:
Symptom control is difficult and/or if there are difficult communication issues or you need advice or support regarding your care delivery supported by the LCP

© Marie Curie Palliative Care Institute Liverpool (MCPCIL)

Name:... NHS no:.. Date:...

Healthcare professional documenting the MDT decision

Following a full MDT assessment and a decision to use the LCP:

Date LCP commenced:...

Time LCP commenced:..

Name (Print):...   Signature:...................................

This will vary according to circumstances and local governance arrangements. In general this should be the most senior healthcare professional immediately available.

The decision must be endorsed by the most senior healthcare professional responsible for the patient's care at the earliest opportunity if different from above.

Name (Print):...   Signature:...................................

All personnel completing the LCP please sign below

You should also have read and understood the guidance on pages 1 - 2

Name (print)	*Full signature*	*Initials*	*Professional title*	*Date*

Record all full MDT reassessments here (including full formal MDT reassessment every 3 days)

Reassessment date:..   Reassessment time:..

Reassessment date:..   Reassessment time:..

Reassessment date:..   Reassessment time:..

Reassessment date:..   Reassessment time:..

If the LCP is discontinued please record here:

Date LCP discontinued..   Time LCP discontinued..

Reasons why the LCP was discontinued:..

....

....

Decision to discontinue the LCP shared with the patient   **Yes ☐   No ☐**

Decision to discontinue the LCP shared with the relative or carer   **Yes ☐   No ☐**

© Marie Curie Palliative Care Institute Liverpool (MCPCIL)

Name:…………………………………………………………… NHS no:………………………………………….. Date:…………………………………

Section 1 Initial assessment (joint assessment by doctor and nurse)

Diagnosis & Baseline Information

DIAGNOSIS:……………………………………………………………………… Co-morbidity:………………………………………………………………………………
……. Ethnicity:……………………………
DOB:……………………………………… Age:………. NHS no:……………………………………………… Female ☐ Male ☐

At the time of the assessment is the patient:

In pain	**Yes** ☐ **No** ☐	Able to swallow	**Yes** ☐ **No** ☐		Confused	**Yes** ☐ **No** ☐
Agitated	**Yes** ☐ **No** ☐	Continent (bladder)	**Yes** ☐ **No** ☐		*(record below which is applicable)*	
Nauseated	**Yes** ☐ **No** ☐	Catheterised	**Yes** ☐ **No** ☐		Conscious	☐
Vomiting	**Yes** ☐ **No** ☐	Continent (bowels)	**Yes** ☐ **No** ☐		Semi-conscious	☐
Dyspnoeic	**Yes** ☐ **No** ☐	Constipated	**Yes** ☐ **No** ☐		Unconscious	☐
Experiencing respiratory tract secretions			**Yes** ☐ **No** ☐			
Experiencing other symptoms (e.g. oedema, itch) ………………………………………………………………………………………………			**Yes** ☐ **No** ☐			

Communication

Goal 1.1: The patient is able to take a full and active part in communication
Achieved ☐ **Variance** ☐ **Unconscious** ☐

Barriers that have the potential to prevent communication have been assessed
First language………………………………… Other issues identified…………………………………………………
Consider need for an interpreter: (contact no) ……………………………………………………………………………..
Other barriers to communication……………………………………………………………………………………………….
Consider: Hearing, vision, speech, learning disabilities, dementia (use of assessment tools), neurological conditions and confusion
The relative or carer may know how specific signs indicate distress if the patient is unable to articulate their own concerns
Does the patient have:-
An advance care plan?
An expressed wish for organ/tissue donation?
An advance decision to refuse treatment (ADRT)?
Does the patient have the capacity to make their own decisions on their own treatment at this moment in time?
consider the support of an IMCA – if required document below:
Comments:……
……...

Goal 1.2: The relative or carer is able to take a full and active part in communication **Achieved** ☐ **Variance** ☐
First language………………………………… Other Issues identified…………………………………………………
Consider need for an interpreter (contact no):…………………………………………………………………………….
Other barriers to communication:…………………………………………………………………………………………….

Goal 1.3: The patient is aware that they are dying **Achieved** ☐ **Variance** ☐ **Unconscious** ☐

Goal 1.4: The relative or carer is aware that the patient is dying **Achieved** ☐ **Variance** ☐

Goal 1.5: The Clinical team have up to date contact information for the relative or carer as documented below
Achieved ☐ **Variance** ☐

1st contact name:…….

Relationship to the patient:………………………………………. Tel no:…………………………. Mobile no:…………………………………
When to contact: At any time ☐ Not at night-time ☐ Staying with the patient overnight ☐

2nd contact:………

Relationship to the patient:………………………………………. Tel no:…………………………. Mobile no:…………………………………
When to contact: At any time ☐ Not at night-time ☐ Staying with patient the overnight ☐

Next of kin - this may be different from above N/A ☐
Name:……………………………………………………………………………………
Contact details:………………………………………………………………………
……………………………………………………………………………………………….
……………………………………………………………………………………………….

Lasting Power of Attorney (LPA) (if applicable) N/A ☐
Name:……………………………………………………………………………
Contact details:……………………………………………………………….
……………………………………………………………………………………………
……………………………………………………………………………………………

© Marie Curie Palliative Care Institute Liverpool (MCPCIL)

Name:………………………………………………………… NHS no:………………………………………….. Date:…………………………………

Section 1 Initial assessment (joint assessment by doctor and nurse)

Facilities

Goal 2: The relative or carer has had a full explanation of the facilities available to them and a facilities leaflet has been given **Achieved ☐ Variance ☐**

Facilities may include: car parking, toilet, bathroom facilities, beverages, payphone, accommodation

Eg. Community Setting - In the patient's own home this could include access details to the district nursing team, palliative care team , out of hours services, GP, home loans, what to do in an emergency, oxygen supplies

Spirituality

Goal 3.1: The patient is given the opportunity to discuss what is important to them at this time eg. their wishes, feelings, faith , beliefs, values **Achieved ☐ Variance ☐ Unconscious ☐**

Patient may be anxious for self or others. Consider specific religious and cultural needs

Consider music, art, poetry, reading, photographs, something that has been important to the belief system or the well-being of the patient

Did the patient take the opportunity to discuss the above **Yes ☐ No ☐ Unconscious ☐**

Religious tradition identified, please specify: ………………………………………………………

Support of the chaplaincy team offered **Yes ☐ No ☐**

If no give reason:………

In-house support Tel/bleep no: ………………………………Name: ……………………………………………… Date/time: ……………………

External support Tel/bleep no: ………………………………Name: ……………………………………………… Date/time: ……………………

Needs now:……..

………

………

………

………

Needs at death:………

………

………

Needs after death:………

………

………

Goal 3.2: The relative or carer is given the opportunity to discuss what is important to them at this time e.g. their wishes, feelings, faith, beliefs, values **Achieved ☐ Variance ☐**

Comments………

………

………

Did the relative or carer take the opportunity to discuss the above **Yes ☐ No ☐**

Medication

Goal 4.1: The patient has medication prescribed on a prn basis for all of the following 5 symptoms which may develop in the last hours or days of life **Achieved ☐ Variance ☐**

Pain ☐
Agitation ☐
Respiratory tract secretions ☐
Nausea / Vomiting ☐
Dyspnoea ☐

Anticipatory prescribing in this manner will ensure that there is no delay in responding to a symptom if it occurs

Current Medication assessed and non essentials discontinued

Medicines for symptom control will only be given when needed, at the right time and just enough and no more than is needed to help the symptom **See Appendix 1 for prescribing guidelines**

Goal 4.2: Equipment is available for the patient to support a continuous subcutaneous infusion (CSCI) of medication where required

Achieved ☐ Variance ☐ Already in place ☐ Not required ☐

If a CSCI is to be used explain the rationale to the patient, relative or carer. Not all patients who are dying will require a CSCI

© Marie Curie Palliative Care Institute Liverpool (MCPCIL)

Name:.. NHS no:.. Date:..

Section 1 Initial assessment (joint assessment by doctor and nurse)

Current Interventions

Goal 5.1: The patient's need for current interventions has been reviewed by the MDT **Achieved ☐ Variance ☐**

	Currently not being taken/ or given	Discontinued	Continued	Commenced
**5a: Routine blood tests**	☐	☐	☐	
**5b: Intravenous antibiotics**	☐	☐	☐	☐
**5c: Blood glucose monitoring**	☐	☐	☐	
**5d: Recording of routine vital signs**	☐	☐	☐	
**5e: Oxygen therapy**	☐	☐	☐	☐

5.2: The patient has a Do Not Attempt Cardiopulmonary Resuscitation Order in place **Achieved ☐ Variance ☐**

Please complete the appropriate associated documentation according to policy and procedure
Explain to the patient, relative or carer as appropriate

5.3: Implantable Cardioverter Defibrillator (ICD) is deactivated **Achieved ☐ Variance ☐ No ICD in place ☐**

Contact the patient's cardiologist. Refer to the ECG technician & refer to local/ regional - policy/procedure.
Information leaflet given to the patient, relative or carer as appropriate

Nutrition

Goal 6: The need for clinically assisted (artificial) nutrition is reviewed by the MDT **Achieved ☐ Variance ☐**

The patient should be supported to take food by mouth for as long as tolerated
For many patients the use of clinically assisted (artificial) nutrition will not be required
A reduced need for food is part of the normal dying process
If clinically assisted (artificial) nutrition is already in place please record route NG ☐ PEG/PEJ ☐ NJ ☐ TPN ☐
Is clinically assisted (artificial) nutrition Not required ☐ Discontinued ☐ Continued ☐
Consider reduction in rate / volume according to individual need if nutritional support is in place
Explain the plan of care to the patient where appropriate, and to the relative or carer

Hydration

Goal 7: The need for clinically assisted (artificial) hydration is reviewed by the MDT **Achieved ☐ Variance ☐**

The patient should be supported to take fluids by mouth for as long as tolerated
For many patients the use of clinically assisted (artificial) hydration will not be required
A reduced need for fluids is part of the normal dying process
Symptoms of thirst / dry mouth do not always indicate dehydration but are often due to mouth breathing or medication. Good mouth care is essential
If clinically assisted (artificial) hydration is already in place please record route IV ☐ S/C ☐ PEG/PEJ ☐ NG ☐
Is clinically assisted (artificial) hydration Not required ☐ Discontinued ☐ Continued ☐ Commenced ☐
Consider reduction in rate / volume according to individual need if hydration support is in place. If required consider the s/c route
Explain the plan of care to the patient where appropriate, and the relative or carer

Skin Care

Goal 8: The patient's skin integrity is assessed **Achieved ☐ Variance ☐**

The aim is to prevent pressure ulcers or further deterioration if a pressure ulcer is present. Use a recognised risk assessment tool e.g. Waterlow / Braden to support clinical judgement. The frequency of repositioning should be determined by skin inspection, assessment and the patient's individual needs. Consider the use of special aids (mattress / bed)
Record the plan of care on the initial assessment MDT sheet where appropriate

Explanation of the plan of care

Goal 9.1: A full explanation of the current plan of care (LCP) is given to the patient
Achieved ☐ Variance ☐ Unconscious☐

Goal 9.2: A full explanation of the current plan of care (LCP) is given to the relative or carer
Achieved ☐ Variance ☐

Name of relative or carer(s) present and relationship to the patient:..
....
Names of healthcare professionals present:...
Information sheet at front of the LCP or equivalent relative or carer information leaflet given Yes ☐ No ☐
Parents or carer should be given or have access to age appropriate advice and information to support children/adolescents

Goal 9.3: The LCP Coping with dying leaflet or equivalent is given to the relative or carer
Achieved ☐ Variance ☐

Goal 9.4: The patient's primary health care team / GP practice is notified that the patient is dying
Achieved ☐ Variance ☐

G.P practice to be contacted if unaware that the patient is dying, message can be left or sent via a secure fax

If you have recorded a variance against any of the goals of care please record on the variance sheet, see page 8

© Marie Curie Palliative Care Institute Liverpool (MCPCIL)

Name:.. NHS no:.. Date:...

**Section 1**	***Initial assessment***	
Signatures	**Please sign here on completion of the initial assessment**	
	**Doctor's name (print):..........................**<br><br>**Doctor's signature:...............................**<br><br>**Date.................Time......................**	**Nurse's name (print):............................**<br><br>**Nurse's signature:................................**<br><br>**Date........................ Time......................**

Section 1 Initial assessment **MDT progress notes**

**Date**	***Supportive information: Plan of care to monitor skin integrity, nutrition / hydration - include here any specific information regarding this patient; relative or carer that has not been captured in the initial assessment that you believe needs to be highlighted.***

© Marie Curie Palliative Care Institute Liverpool (MCPCIL)

Name:... NHS no:... Date:...

Variance analysis sheet for initial assessment

What variance occurred & why? (what was the issue?)	Action taken (what did you do?)	Outcome (did this solve the issue?)
Goal: <br>***Signature:..*** ***Date / Time:....................................***	***Signature:.......................................*** ***Date / Time:....................................***	***Signature:..*** ***Date / Time:....................................***
Goal: <br>***Signature:..*** ***Date / Time:....................................***	***Signature:..*** ***Date / Time:....................................***	***Signature:..*** ***Date / Time:....................................***
Goal: <br>***Signature:..*** ***Date / Time:....................................***	***Signature:..*** ***Date / Time:....................................***	***Signature:..*** ***Date / Time:....................................***
Goal: <br>***Signature:..*** ***Date / Time:....................................***	***Signature:..*** ***Date / Time:....................................***	***Signature:..*** ***Date / Time:....................................***
Goal: <br>***Signature:..*** ***Date / Time:....................................***	***Signature:..*** ***Date / Time:....................................***	***Signature:..*** ***Date / Time:....................................***

© Marie Curie Palliative Care Institute Liverpool (MCPCIL)

Name:…………………………………………………………… NHS no:…………………………………………….. Date:…………………………………

Section 2 Ongoing assessment of the plan of care – LCP DAY……..

Undertake an MDT assessment & review of the current management plan if:

Improved conscious level, functional ability, oral intake, mobility, ability to perform self-care

and or

Concern expressed regarding management plan from either the patient, relative or team member

and or

It is 3 days since the last **full** MDT assessment

Consider the support of the specialist palliative care team and/or a second opinion as required. Document all reassessment dates and times on page 3

Codes to be recorded at each timed assessment (a moment in time) A= Achieved V = Variance (exception reporting)

Record an A or a V not a signature	0400	0800	1200	1600	2000	2400
**Goal a: The patient does not have pain**<br>Verbalised by patient if conscious, pain free on movement. Observe for non-verbal cues. Consider need for positional change. Use a pain assessment tool if appropriate. Consider prn analgesia for incident pain						
**Goal b: The patient is not agitated**<br>Patient does not display signs of restlessness or distress, exclude reversible causes e.g. retention of urine, opioid toxicity						
**Goal c: The patient does not have respiratory tract secretions**<br>Consider positional change. Discuss symptoms & plan of care with patient, relative or carer<br>Medication to be given as soon as symptom occurs						
**Goal d: The patient does not have nausea**<br>Verbalised by patient if conscious						
**Goal e: The patient is not vomiting**						
**Goal f: The patient is not breathless**<br>Verbalised by patient if conscious, consider positional change. Use of a fan may be helpful						
**Goal g: The patient does not have urinary problems**<br>Use of pads, urinary catheter as required						
**Goal h: The patient does not have bowel problems**<br>Monitor – constipation / diarrhoea. Monitor skin integrity<br>Bowels last opened:………………………						
**Goal i: The patient does not have other symptoms**<br>Record symptom here……………………………………………………….<br>*If no other symptoms present please record N/A*						
**Goal j: The patient's comfort & safety regarding the administration of medication is maintained**<br>If CSCI in place – monitoring sheet in progress<br>S/C butterfly in place if needed for prn medication location:………………………….<br>The patient is only receiving medication that is beneficial at this time. *If no medication required please record N/A*						

© Marie Curie Palliative Care Institute Liverpool (MCPCIL)

Name:…………………………………………………………… NHS no:………………………………………….. Date:…………………………………

Section 2 Ongoing assessment of the plan of care – LCP continued DAY….

Codes to be recorded at each timed assessment (a moment in time) A= Achieved V = Variance (exception reporting)

	0400	0800	1200	1600	2000	2400
**Goal k: The patient receives fluids to support their individual needs**<br>The patient is supported to take oral fluids / thickened fluids for as long as tolerated. Monitor for signs of aspiration and/or distress. If symptomatically dehydrated & not deemed futile, consider clinically assisted (artificial) hydration if in the patient's best interest. If in place monitor & review rate/volume. Explain the plan of care with the patient and relative or carer						
**Goal l: The patient's mouth is moist and clean**<br>See mouth care policy. Relative or carer involved in care giving as appropriate. Mouth care tray at the bedside						
**Goal m: The patient's skin integrity is maintained**<br>Assessment, cleansing, positioning, use of special aids (mattress / bed). The frequency of repositioning should be determined by skin inspection and the patient's individual needs. *Waterlow / Braden score:…………………*						
**Goal n: The patient's personal hygiene needs are met**<br>Skin care, wash, eye care, change of clothing according to individual needs. Relative or carer involved in care giving as appropriate						
**Goal o: The patient receives their care in a physical environment adjusted to support their individual needs**<br>Well fitting curtains, screens, clean environment, sufficient space at bedside, consider fragrance, silence, music, light, dark, pictures, photographs, nurse call bell accessible						
**Goal p: The patient's psychological well-being is maintained**<br>Staff just being at the bedside can be a sign of support and caring. Respectful verbal and non-verbal communication, use of listening skills, information and explanation of care given. Use of touch if appropriate. Spiritual/religious/cultural needs – consider support of the chaplaincy team						
**Goal q: The well-being of the relative or carer attending the patient is maintained**<br>Just being at the bedside can be a sign of support and caring. Consider spiritual/religious/cultural needs, expressions may be unfamiliar to the healthcare professional but normal for the relative or carer – support of chaplaincy team may be helpful. Listen & respond to worries/fears. Age appropriate advice & information to support children/adolescents available to parents or carers. Allow the opportunity to reminisce. Offer a drink						
**Signature of the person making the assessment**						
**Signature of the registered nurse per shift**	**Night**	**Early**		**Late**		**Night**

© Marie Curie Palliative Care Institute Liverpool (MCPCIL)

Name:…………………………………………………………… NHS no:………………………………………….. Date:……………………………….

Section 2 Ongoing assessment of the plan of care – LCP DAY……..

Undertake an MDT assessment & review of the current management plan if:

Improved conscious level, functional ability, oral intake, mobility, ability to perform self-care	**and or**	Concern expressed regarding management plan from either the patient, relative or team member	**and or**	It is 3 days since the last **full** MDT assessment

Consider the support of the specialist palliative care team and/or a second opinion as required. Document all reassessment dates and times on page 3

Codes to be recorded at each timed assessment (a moment in time) A= Achieved V = Variance (exception reporting)

**Record an A or a V not a signature**	**0400**	**0800**	**1200**	**1600**	**2000**	**2400**
**Goal a: The patient does not have pain**<br>Verbalised by patient if conscious, pain free on movement. Observe for non-verbal cues. Consider need for positional change. Use a pain assessment tool (**see Appendix 2**) if appropriate. Consider prn analgesia for incident pain						
**Goal b: The patient is not agitated**<br>Patient does not display signs of restlessness or distress, exclude reversible causes e.g. retention of urine, opioid toxicity						
**Goal c: The patient does not have respiratory tract secretions**<br>Consider positional change. Discuss symptoms & plan of care with patient, relative or carer<br>Medication to be given as soon as symptom occurs						
**Goal d: The patient does not have nausea**<br>Verbalised by patient if conscious						
**Goal e: The patient is not vomiting**						
**Goal f: The patient is not breathless**<br>Verbalised by patient if conscious, consider positional change. Use of a fan may be helpful						
**Goal g: The patient does not have urinary problems**<br>Use of pads, urinary catheter as required						
**Goal h: The patient does not have bowel problems**<br>Monitor – constipation / diarrhoea. Monitor skin integrity<br>Bowels last opened:……………………..						
**Goal i: The patient does not have other symptoms**<br>Record symptom here……………………………………………………….<br>*If no other symptoms present please record N/A*						
**Goal j: The patient's comfort & safety regarding the administration of medication is maintained**<br>If CSCI in place – monitoring sheet in progress<br>S/C butterfly in place if needed for prn medication location:………………………..<br>The patient is only receiving medication that is beneficial at this time. *If no medication required please record N/A*						

© Marie Curie Palliative Care Institute Liverpool (MCPCIL)

**Section 2 Ongoing assessment of the plan of care – LCP continued DAY….**						
Codes to be recorded at each timed assessment (a moment in time) A= Achieved V = Variance (exception reporting)						
	**0400**	**0800**	**1200**	**1600**	**2000**	**2400**
**Goal k: The patient receives fluids to support their individual needs**<br>The patient is supported to take oral fluids / thickened fluids for as long as tolerated. Monitor for signs of aspiration and/or distress. If symptomatically dehydrated & not deemed futile, consider clinically assisted (artificial) hydration if in the patient's best interest. If in place monitor & review rate/volume. Explain the plan of care with the patient and relative or carer						
**Goal l: The patient's mouth is moist and clean**<br>See mouth care policy. Relative or carer involved in care giving as appropriate. Mouth care tray at the bedside						
**Goal m: The patient's skin integrity is maintained**<br>Assessment, cleansing, positioning, use of special aids (mattress / bed). The frequency of repositioning should be determined by skin inspection and the patient's individual needs. *Waterlow / Braden score:…………………*						
**Goal n: The patient's personal hygiene needs are met**<br>Skin care, wash, eye care, change of clothing according to individual needs. Relative or carer involved in care giving as appropriate						
**Goal o: The patient receives their care in a physical environment adjusted to support their individual needs**<br>Well fitting curtains, screens, clean environment, sufficient space at bedside, consider fragrance, silence, music, light, dark, pictures, photographs, nurse call bell accessible						
**Goal p: The patient's psychological well-being is maintained**<br>Staff just being at the bedside can be a sign of support and caring. Respectful verbal and non-verbal communication, use of listening skills, information and explanation of care given. Use of touch if appropriate. Spiritual/religious/cultural needs – consider support of the chaplaincy team						
**Goal q: The well-being of the relative or carer attending the patient is maintained**<br>Just being at the bedside can be a sign of support and caring. Consider spiritual/religious/cultural needs, expressions may be unfamiliar to the healthcare professional but normal for the relative or carer – support of chaplaincy team may be helpful. Listen & respond to worries/fears. Age appropriate advice & information to support children/adolescents available to parents or carers. Allow the opportunity to reminisce. Offer a drink						
**Signature of the person making the assessment**						
**Signature of the registered nurse per shift**	**Night**	**Early**		**Late**		**Night**

© Marie Curie Palliative Care Institute Liverpool (MCPCIL)

Name:…………………………………………………………… NHS no:…………………………………………….. Date:…………………………………

Section 2 Ongoing assessment of the plan of care – LCP DAY……..

Undertake an MDT assessment & review of the current management plan if:

Improved conscious level, functional ability, oral intake, mobility, ability to perform self-care

and or

Concern expressed regarding management plan from either the patient, relative or team member

and or

It is 3 days since the last **full** MDT assessment

Consider the support of the specialist palliative care team and/or a second opinion as required. Document all reassessment dates and times on page 3

Codes to be recorded at each timed assessment (a moment in time) A= Achieved V = Variance (exception reporting)

**Record an A or a V not a signature**	**0400**	**0800**	**1200**	**1600**	**2000**	**2400**
**Goal a: The patient does not have pain**<br>Verbalised by patient if conscious, pain free on movement. Observe for non-verbal cues. Consider need for positional change. Use a pain assessment tool if appropriate. Consider prn analgesia for incident pain						
**Goal b: The patient is not agitated**<br>Patient does not display signs of restlessness or distress, exclude reversible causes e.g. retention of urine, opioid toxicity						
**Goal c: The patient does not have respiratory tract secretions**<br>Consider positional change. Discuss symptoms & plan of care with patient, relative or carer<br>Medication to be given as soon as symptom occurs						
**Goal d: The patient does not have nausea**<br>Verbalised by patient if conscious						
**Goal e: The patient is not vomiting**						
**Goal f: The patient is not breathless**<br>Verbalised by patient if conscious, consider positional change. Use of a fan may be helpful						
**Goal g: The patient does not have urinary problems**<br>Use of pads, urinary catheter as required						
**Goal h: The patient does not have bowel problems**<br>Monitor – constipation / diarrhoea. Monitor skin integrity<br>Bowels last opened:……………………..						
**Goal i: The patient does not have other symptoms**<br>Record symptom here……………………………………………………….<br>*If no other symptoms present please record N/A*						
**Goal j: The patient's comfort & safety regarding the administration of medication is maintained**<br>If CSCI in place – monitoring sheet in progress<br>S/C butterfly in place if needed for prn medication location:………………………..<br>The patient is only receiving medication that is beneficial at this time. *If no medication required please record N/A*						

© Marie Curie Palliative Care Institute Liverpool (MCPCIL)

Name:………………………………………………………… NHS no:…………………………………………. Date:…………………………………

Section 2 Ongoing assessment of the plan of care – LCP continued DAY….

Codes to be recorded at each timed assessment (a moment in time) A= Achieved V = Variance (exception reporting)

	0400	0800	1200	1600	2000	2400
**Goal k: The patient receives fluids to support their individual needs**<br>The patient is supported to take oral fluids / thickened fluids for as long as tolerated. Monitor for signs of aspiration and/or distress. If symptomatically dehydrated & not deemed futile, consider clinically assisted (artificial) hydration if in the patient's best interest. If in place monitor & review rate/volume. Explain the plan of care with the patient and relative or carer						
**Goal l: The patient's mouth is moist and clean**<br>See mouth care policy. Relative or carer involved in care giving as appropriate. Mouth care tray at the bedside						
**Goal m: The patient's skin integrity is maintained**<br>Assessment, cleansing, positioning, use of special aids (mattress / bed). The frequency of repositioning should be determined by skin inspection and the patient's individual needs. *Waterlow / Braden score:…………………*						
**Goal n: The patient's personal hygiene needs are met**<br>Skin care, wash, eye care, change of clothing according to individual needs. Relative or carer involved in care giving as appropriate						
**Goal o: The patient receives their care in a physical environment adjusted to support their individual needs**<br>Well fitting curtains, screens, clean environment, sufficient space at bedside, consider fragrance, silence, music, light, dark, pictures, photographs, nurse call bell accessible						
**Goal p: The patient's psychological well-being is maintained**<br>Staff just being at the bedside can be a sign of support and caring. Respectful verbal and non-verbal communication, use of listening skills, information and explanation of care given. Use of touch if appropriate. Spiritual/religious/cultural needs – consider support of the chaplaincy team						
**Goal q: The well-being of the relative or carer attending the patient is maintained**<br>Just being at the bedside can be a sign of support and caring. Consider spiritual/religious/cultural needs, expressions may be unfamiliar to the healthcare professional but normal for the relative or carer – support of chaplaincy team may be helpful. Listen & respond to worries/fears. Age appropriate advice & information to support children/adolescents available to parents or carers. Allow the opportunity to reminisce. Offer a drink						
**Signature of the person making the assessment**						
**Signature of the registered nurse per shift**	**Night**	**Early**		**Late**		**Night**

© Marie Curie Palliative Care Institute Liverpool (MCPCIL)

Name:…………………………………………………………… NHS no:…………………………………………….. Date:…………………………………

Section 2 Ongoing assessment MDT progress notes

Date/time	Record significant events/conversations/medical review/visit by other specialist teams e.g. palliative care / second opinion if sought	Signature

© Marie Curie Palliative Care Institute Liverpool (MCPCIL)

Name:………………………………………………………… NHS no:…………………………………………… Date:…………………………………

Section 2 Ongoing assessment MDT progress notes

Date / time	Record significant events/conversations/medical review/visit by other specialist teams e.g. palliative care/second opinion if sought	Signature

© Marie Curie Palliative Care Institute Liverpool (MCPCIL)

Name:…………………………………………………………… NHS no:………………………………………….. Date:…………………………………

Section 3 Care after death

Verification of death

Time of the patient's death recorded by the healthcare professional in the organisation:………………………………………………..
Date of patient's death: ………/………/………
Verified by doctor ☐ Verified by senior nurse ☐ Date / time verified:……………………………………………………………………
Cause of death……..
Details of healthcare professional who verified death
Name:…………………………………………………………………… (please print) Signature:…………………………………………… Bleep No:……………………..
Comments:………
……….
Persons present at time of death:……….
Relative or carer present at time of death: Yes ☐ No ☐ If not present, have the relative or carer been notified Yes ☐ No ☐
Name of person informed:……………………………………………………… Relationship to the patient:……………………………………………………………..
Contact number:…………………………………………………………………………
Is the coroner likely to be involved: Yes ☐ No ☐
Consultant /GP:……………………..………………… Doctor:…………………………………………… Bleep No:………….. Tel No:…………………………………

Patient Care Dignity	**Goal 10: last offices are undertaken according to policy and procedure** **Achieved** ☐ **Variance** ☐<br>The patient is treated with respect and dignity whilst last offices are undertaken<br>Universal precautions & local policy and procedures including infection risk adhered to<br>Spiritual, religious, cultural rituals / needs met<br>Organisational policy followed for the management of ICDs, where appropriate<br>Organisational policy followed for the management & storage of patient's valuables and belongings
**Relative or Carer Information**	**Goal 11: The relative or carer can express an understanding of what they will need to do next and are given relevant written information** **Achieved** ☐ **Variance** ☐<br>Conversation with relative or carer explaining the next steps<br>Grieving leaflet given Yes ☐ No ☐<br>DWP1027 (England & Wales) or equivalent is given Yes ☐ No ☐<br>Information given regarding how and when to contact the bereavement office / general office / funeral director to make an appointment – regarding the death certificate and patient's valuables and belongings where appropriate<br>Wishes regarding tissue/organ donation discussed<br>Discuss as appropriate: viewing the body / the need for a post mortem / the need for removal of cardiac devices / the need for a discussion with the coroner<br>Information given to families on child bereavement services where appropriate – national & local agencies
**Organisation Information**	**Goal 12.1: The primary health care team / GP is notified of the patient's death** **Achieved** ☐ **Variance** ☐<br>The primary health care team / GP may have known this patient very well and other relatives or carers may be registered with the same GP Telephone or fax the GP practice
	**Goal 12.2: The patient's death is communicated to appropriate services across the organisation** **Achieved** ☐ **Variance** ☐<br>e.g. Bereavement office / general office / palliative care team / district nursing team / community matron (where appropriate) are informed of the death<br>The patient's death is entered on the organisation's IT system

Healthcare professional signature:…………………………………………………………
Date:……………………………………… Time:…………………………………………

Please record any variance on the variance sheet overleaf

Section 3 Care after death MDT progress notes - record any significant issues not reflected above

Date	

© Marie Curie Palliative Care Institute Liverpool (MCPCIL)

Name:………………………………………………………… NHS no:…………………………………………. Date:…………………………………

Variance analysis sheet for section 2 and 3 of the LCP		
**What variance occurred & why? (what was the issue?)**	**Action taken (what did you do?)**	**Outcome (did this solve the issue?)**
Goal:<br><br>***Signature:……………………………………***<br>***Date / Time:…………………………………***	***Signature:……………………………………***<br>***Date / Time:…………………………………***	***Signature:……………………………………***<br>***Date / Time:…………………………………***
Goal:<br><br>***Signature:……………………………………***<br>***Date / Time:…………………………………***	***Signature:……………………………………***<br>***Date / Time:…………………………………***	***Signature:……………………………………***<br>***Date / Time:…………………………………***
Goal:<br><br>***Signature:……………………………………***<br>***Date / Time:…………………………………***	***Signature:……………………………………***<br>***Date / Time:…………………………………***	***Signature:……………………………………***<br>***Date / Time:…………………………………***
Goal:<br><br>***Signature:……………………………………***<br>***Date / Time:…………………………………***	***Signature:……………………………………***<br>***Date / Time:…………………………………***	***Signature:……………………………………***<br>***Date / Time:…………………………………***
Goal:<br><br>***Signature:……………………………………***<br>***Date / Time:…………………………………***	***Signature:……………………………………***<br>***Date / Time:…………………………………***	***Signature:……………………………………***<br>***Date / Time:…………………………………***

© Marie Curie Palliative Care Institute Liverpool (MCPCIL)

Name:.. NHS no:... Date:..

Variance analysis sheet for section 2 and 3 of the LCP		
**What variance occurred & why? (what was the issue?)**	**Action taken (what did you do?)**	**Outcome (did this solve the issue?)**
Goal: <br>***Signature:...*** ***Date / Time:....................................***	***Signature:...*** ***Date / Time:....................................***	***Signature:...*** ***Date / Time:....................................***
Goal: <br>***Signature:...*** ***Date / Time:....................................***	***Signature:...*** ***Date / Time:....................................***	***Signature:...*** ***Date / Time:....................................***
Goal: <br>***Signature:...*** ***Date / Time:....................................***	***Signature:...*** ***Date / Time:....................................***	***Signature:...*** ***Date / Time:....................................***
Goal: <br>***Signature:...*** ***Date / Time:....................................***	***Signature:...*** ***Date / Time:....................................***	***Signature:...*** ***Date / Time:....................................***

LCP SUPPORTING INFORMATION

Each organisation must develop medication guidance in accordance with local medicines management / palliative care guidelines / policy & procedure and reference them accordingly.

It is helpful to have the guidance attached to each LCP.

© Marie Curie Palliative Care Institute Liverpool (MCPCIL)

Appendix 1

As required and breakthrough medication prescriptions

Assess Symptoms – Prescribe anticipatory medications

- Pain/dyspnoea – Diamorphine – if opioid naïve 1.25 – 2.5mg by subcutaneous bolus or 5 – 10mgs in 24 hours by continuous subcutaneous infusion. Ensure breakthrough dose is 1/6 of total daily dose prescribed. Sufficient parenteral opioid (relative to the background oral / driver dose) should be available for breakthrough for those who are already on opioid's.

- Nausea / Agitation – Levomepromazine 6.25 – 10mg by subcutaneous bolus or up to 200mg in 24 hours by continuous subcutaneous infusion. Low doses should be suitable for nausea but doses may need to be titrated upwards for agitation.

- Restlessness / Anxiety / Fits – Midazolam 2.5 – 10mgs by subcutaneous bolus repeated after 1 hour if necessary or 10 – 80mgs in 24 hours by continuous subcutaneous infusion. Rule out other causes of agitation – urinary retention, pain, constipation.

- Respiratory Tract Secretions – Hyoscine hydrobromide 200-400mcgs by subcutaneous bolus or up to 2.4mgs in 24hours by continuous subcutaneous infusion.

(Adapted from Highland Palliative Care Guidelines)

© Marie Curie Palliative Care Institute Liverpool (MCPCIL)

Appendix 2

Name:.. NHS no:.. Date:...

NHS Eileanan Siar Western Isles

Name:...............................
Chi NO:...............................
Address:..............................
....

PAIN ASSESSMENT FORM—INITIAL ASSESSMENT

Body Map

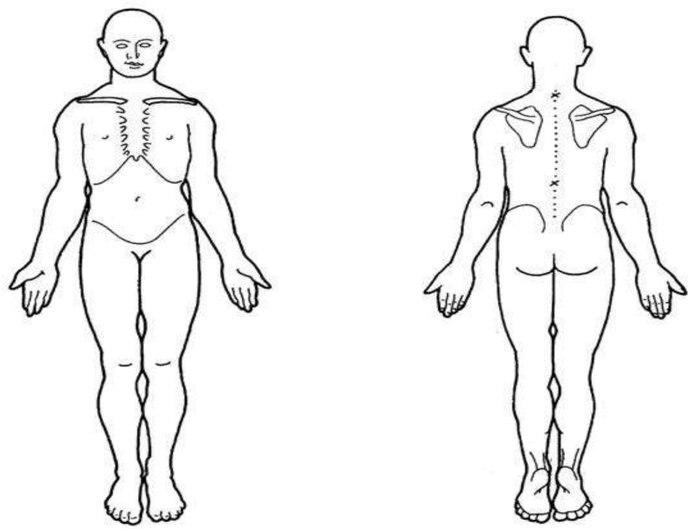

Verbal (circle all that are appropriate)

Burning	Throbbing	Stinging	Sharp
Nagging	Dull ache	Crushing	Gnaw-ing
Cramping	Stabbing	Other........	

LABEL EACH PAIN SITE WITH A B C etc

Visual Analogue Scale

Score	Description
10	Overwhelming
9	Severe pain—disabling, prevents from doing usual activities 7—9
8	
7	
6	Moderate pain limits some activities 4—6
5	
4	
3	Mild pain 1—3
2	
1	
0	No pain

Score your pain by circling the number that best describes your pain at its worst in the past 24 hours.

Assessment

Patients own description of the pain

Is the pain constant or intermittent?

What helps relieve the pain?

What makes the pain worse?

Are there particular times when pain is worse?

Is pain evident on movement?

Is sleep affected?

Is appetite affected?

Is mood affected?

Is current medication effective?

Assessment competed by:

Patient ☐

Patient & Healthcare Professional ☐

Healthcare Professional ☐

(patient unable to co-operate)

SIGNED: ....

REVIEWED BY NURSE/SIGNATURE

....

Name:... NHS no:... Date:...

DAILY PAIN ASSESSMENT

DATE	TIME	PAIN SITE	SCORE	REGULAR ANALGESIA	BREAKTHROUGH ANALGESIA	ASSESSED BY *	EVALUATION	NURSE SIGNATURE	DOCTOR SIGNATURE

Use following key: **P = Patient**

P + H = Patient & Healthcare Professional

HCP = Healthcare Professionals

© Marie Curie Palliative Care Institute Liverpool (MCPCIL)
