# Supplementary material for: The Liverpool Care Pathway for the Dying Patient: a critical analysis of its rise, demise and legacy in England
Source: Wellcome Open Res. 2018 Apr 24;3:15. Originally published 2018 Feb 22. [Version 2] doi: 10.12688/wellcomeopenres.13940.2 (PMC5963294; doi:10.12688/wellcomeopenres.13940.2)
Supplement: Peer review history

# Peer review: Referee response for version 1

**DOI:** 10.21956/wellcomeopenres.15154.r31077

**Published:** 2018-04-06

**Authors:** Richard Huxtable

**Affiliations:** 1 Centre for Ethics in Medicine, Bristol Medical School, University of Bristol, Bristol, UK

In this thoughtful and illuminating article, Professors Seymour and Clark critically analyse the rise, demise and legacy of the Liverpool Care Pathway (LCP), using the sociological concept of “boundary objects”. A boundary object occupies multiple social worlds and enables communication between them, making this a good conceptual fit to the LCP, which was an integrated care pathway that sought to straddle various clinical, managerial and “user” interests.

 The use of the boundary objects metaphor is instructive and the authors (commendably) clearly marshal and convey a diverse range of evidence from the literature. The authors take a literature-based approach, in part because qualitative work (e.g. interviewing key participants and stakeholders) would likely be premature. This seems plausible, although future such work would no doubt be welcome. It might have been useful to hear a little more about how the authors designed their (systematic?) review, but this certainly appears to be a thorough review, which successfully traces the history of the LCP and highlights the key themes surrounding the LCP that one might expect to encounter.

 Numerous overlapping themes emerge: striking a balance between individualised (person-centred) care and standardised care; the nature and use of evidence in supporting or discrediting health interventions (and, indeed, in policy-making); translating theory into practice, through appropriate education, support and dissemination; and the respective roles of process and judgment when designing and delivering care.

 Many of the themes will resonate beyond the LCP and the authors do engage with some (important) broader themes, such as the role of care pathways, funding of and in the UK’s National Health Service, and perceptions of palliative and end of life care more generally. Some such themes might even have been pushed further e.g. in spurring national discussions about death and dying, might the LCP’s demise be associated with an oft-reported reluctance to openly confront these difficult matters? However, the authors’ reflections appear to be carefully evidence-based. Indeed, the evidential issues surrounding the LCP were notable throughout their article: I was particularly struck by the sense that the sort of evidence that was judged problematically to underpin the LCP – this being “soft”, less than “gold standard”, evidence – was precisely the sort of evidence then used to discredit it, and to underpin its review and subsequent removal. The sort of hard evidence sought by some critics was later collected, but I was left pondering the sort of evidence best suited to this complex, uncertain context (indeed, uncertainty might be another theme associated with the LCP, as the authors acknowledge early in their article).

 In discussing the themes that they found, the authors display a sure grasp of context and history. Some elements could have been explained a little further, especially for non-UK audiences and for those less familiar with navigating the (potentially bewildering) health policy landscape. As other reviewers also comment, examples include the references to the Gold Standard Framework, Preferred Priorities of Care, End of Life Care Strategy, and to bodies like the National Council for Palliative Care (which has since merged with Hospice UK). There are also very occasional – and very mild – typographical errors. However, none of this should detract from the value of this important article. The authors display insight and balance throughout and this article should become a go-to piece for anyone wishing to explore the LCP and, more generally, the promise and pitfalls of using pathways and making policy in healthcare.

I have read this submission. I believe that I have an appropriate level of expertise to confirm that it is of an acceptable scientific standard.

# Response

**Published:** 2018-04-20

**Authors:** David Clark

**Affiliations:** University of Glasgow, UK

We thank Professor Huxtable for his insightful review. Work is underway to collect interview data from key stakeholders involved in the international application of the LCP in a follow on project. The latter will provide us with an opportunity to consider some of the important issues raised in this review, such as the relationship between palliative care policy and wider societal perceptions of end of life care, and the type of ‘evidence’ required to underpin innovations in palliative care. We have made some small clarifications to the paper, which hopefully eradicate the typographical errors identified and make aspects of the paper a little more accessible to international readers.

# Peer review: Referee response for version 1

**DOI:** 10.21956/wellcomeopenres.15154.r31075

**Published:** 2018-03-29

**Authors:** Joakim Ohlen, Cecilia Håkanson

**Affiliations:** 1 Institute of Health and Care Sciences, University of Gothenburg Centre for Person-Centred Care, Sahlgrenska Academy at the University of Gothenburg, Gothenburg, Sweden; 2 Department of Nursing Science, Sophiahemmet University, Stockholm, Sweden

The story of success and subsequent controversy surrounding the Liverpool Care Pathway for the dying (LCP) in United Kingdom is widely known internationally. However, it may not be clear to an international audience just what this story is based on, although it is highly relevant. For this reason, we commend the analysis put forward by Drs Jane Seymour and David Clark. Their historical analysis of publicly available information sources about LCP in Britain is important and contributes by combining critical analysis with a data- and theory-informed understanding of what contributed to the widespread use and implementation of this clinical pathway, how it could rapidly be adopted as a policy, and then legally advised against. Nevertheless, having discussed the presentation of the LCP conflict by the authors in the background, we find the introductory perspective to be somewhat less distanced than the rest of the article.

 The study is contextualized to the global spread of palliative care and how it has evolved as a response to challenges in the population, while the analysis focuses on LCP on the UK scene. International readers might benefit from having British palliative care put in context, within the responsibility of the NHS on the one hand and wider society – and maybe communities – on the other. This particularly surfaces in the analysis, where the authors clarify that, at the same time as the LCP, the ‘Gold Standards Framework’ and the ‘Preferred Priorities for Care’ were also implemented. How these three tools were related is not explicated, however. All three are mentioned, but it remains unclear to what extent, if at all, these latter tools were part of the LCP discourse, and if so, in what way.

 The theoretical lens of ‘boundary objects’ which is used in the study appears to have a practical fit. In our interpretation, the lens articulates – and demarcates – an understanding of the study object that could range along opposites poles in a continuum from considered negative to positive, which is related to negotiations, renegotiations and conflicts as reflected in the swings of the pendulum appearing in the history of the LCP. Moreover, the perspective could help shed light on how different perspectives could be bridged and also on unintended consequences, which were clearly related to the implementation of the LCP. However, the article does not make clear what aspects might have been left out in the analysis. This would have been useful, especially for international readers who are not knowledgeable of the details in the British context. Moreover, the discussion section does little to go back or problematize from the view point of this framework.

 A fairly unsurprising but still important result of the analysis is the fact that the tool itself cannot be blamed for poor quality of care. Thus, the analysis explicates the importance of considering what a practice tool involves (e.g. as related to underpinnings, reliability, usefulness and so forth) and the driving forces for implementation and diffusion of the tool. After independently reading the article and then discussing it, we became unsure as to how the whole controversy were handled by the media – could this have been more explicated and problematized in the analysis? This also raises the question of whether the NHS makes room for self-criticism (i.e. allows sufficient implementation and follow-up procedure, and a workable structure, resources and competence of care etc.).

 On the whole, we find this analysis complete and important, both for practice and research as related to palliative care practice and policy development and, of course, especially for knowledge translation or dissemination. In our view, the significance of the analysis is that it shows how critical it is to have an informed understanding of the not only the development of innovative practice support tools, but moreover of complexities and the diversity of stakeholders involved in their dissemination, and that both innovative development processes and implementation processes do not only happen in discourses of evidenced-based practice. Over simplified conclusions of problems related to the use of several practice tools in palliative care, such as the LCP, are either that the tool has sufficient evidence or that the tool in itself is dangerous or disconcerts wellbeing for patients and/or their informal carers. Furthermore, the analysis of the LCP case shows that a tool can be both recommended and promoted despite lacking support from significant evidence gatekeepers such as Cochrane reviews. Rather, the analysis by Seymour and Clark clarifies the need for practitioners to take action based on their practical wisdom and not only rely on tools, as well as the merits of considering unintended consequences related to the use of tools. At least from our Scandinavian perspective we could learn from the LCP lesson, and without doubts Seymour & Clark’s analysis contributes to a more in-depth and critical understanding than previous national reports from the UK on the use and recommendations of LCP.

We have read this submission. We believe that we have an appropriate level of expertise to confirm that it is of an acceptable scientific standard.

# Response

**Published:** 2018-04-20

**Authors:** David Clark

**Affiliations:** University of Glasgow, UK

We thank Professor Öhlen and Dr Håkanson for their thoughtful and helpful review. We accept their view that the some aspects of the paper (especially the relationship between the three ‘tools’ ) may be challenging for an international audience unfamiliar with the UK context, but hope that the small clarifications we have now made to the paper go some way to addressing this.  We anticipate making further use of the boundary object concept in a follow on study examining the international spread of the LCP, and so their observation that we could perhaps have made further interpretive use of the latter in this paper is valuable to us as we embark on that new project. The observations about what may have been left out of the analysis and whether we could have provided an expanded account to the role of the media are well made. It was challenging to weave together a coherent narrative about a very complex affair and we have tried to be balanced in our use of sources. We hope that the paper will encourage others to take a similarly ‘forensic’ approach to examining the many aspects of this important phenomenon in palliative care history.
